# De novo assembly of the complete mitochondrial genome of sweet potato (*Ipomoea batatas* [L.] Lam) revealed the existence of homologous conformations generated by the repeat-mediated recombination

**DOI:** 10.1186/s12870-022-03665-y

**Published:** 2022-06-10

**Authors:** Zhijian Yang, Yang Ni, Zebin Lin, Liubin Yang, Guotai Chen, Nuerla Nijiati, Yunzhuo Hu, Xuanyang Chen

**Affiliations:** 1 Key Laboratory of Crop Biotechnology, Fujian Agriculture and Forestry University, Fujian Province Universities, Fuzhou, China; 2 College of Agriculture, Fujian Agriculture and Forestry University, Fuzhou, China; 3 Fujian Provincial Key Laboratory of Crop Breeding by Design, Fuzhou, Fujian China

**Keywords:** *Ipomoea batatas*, Mitochondrial genome, De novo assembly, Repeat-mediated recombination, RNA editing events

## Abstract

Sweet potato (*Ipomoea batatas* [L.] Lam) is an important food crop, an excellent fodder crop, and a new type of industrial raw material crop. The lack of genomic resources could affect the process of industrialization of sweet potato. Few detailed reports have been completed on the mitochondrial genome of sweet potato. In this research, we sequenced and assembled the mitochondrial genome of sweet potato and investigated its substructure. The mitochondrial genome of sweet potato is 270,304 bp with 23 unique core genes and 12 variable genes. We detected 279 pairs of repeat sequences and found that three pairs of direct repeats could mediate the homologous recombination into four independent circular molecules. We identified 70 SSRs in the whole mitochondrial genome of sweet potato. The longest dispersed repeat in mitochondrial genome was a palindromic repeat with a length of 915 bp. The homologous fragments between the chloroplast and mitochondrial genome account for 7.35% of the mitochondrial genome. We also predicted 597 RNA editing sites and found that the *rps*3 gene was edited 54 times, which occurred most frequently. This study further demonstrates the existence of multiple conformations in sweet potato mitochondrial genomes and provides a theoretical basis for the evolution of higher plants and cytoplasmic male sterility breeding.

**Supplementary Information:**

The online version contains supplementary material available at 10.1186/s12870-022-03665-y.

## Introduction

Sweet potato [(*Ipomoea batatas* (L.) Lam.], which belongs to the botanical family Convolvulaceae [1], is an important food crop, an excellent fodder crop, and a new type of industrial raw material crop [2, 3]. The Food and Agriculture Organization of the United Nations (FAO) considers sweet potato one of the important crops in solving food shortage and energy problems in the twenty-first century. In 2020, 7.40 million hectares of sweet potato were planted worldwide, with a total production of 89.49 million tons (https://www.fao.org/faostat/zh/#data,2022). The production and storage process of sweet potato is green and pollution-free, which is why sweet potato is considered “the most ideal food in the 21st century” [3]. In addition to being rich in starch, sweet potatoes also contain protein, minerals, and trace elements, especially a large amount of carotenoids and anthocyanins [4, 5]. These compounds have important health functions for human health, and recently, many new varieties of sweet potato with high pigment content have been successfully bred and applied in production [6, 7]. As sweet potatoes can be harvested for fresh consumption or for processing various food products, its preservation and processing technologies have been widely researched to improve the technology and efficiency of the sweet potato industry [8]. Sweet potato production is severely affected by biotic adversities, such as small weevil (weevil), SPVD virus disease, and root rot, as well as abiotic adversities such as drought and salinity [9–11], which can lead to yield losses and reduced quality of sweet potato. In the process of selecting and breeding new varieties with high resistance, many genes for good traits, especially genes for specific traits derived from wild species, are difficult to introduce into new varieties due to the complex genome [12]. With the development of bioinformatics, sweet potato has made great progress in genome, transcriptome, and small RNA analysis, laying the foundation for improving sweet potato traits and ensuring production safety [13–15].

Mitochondria are an essential organelle in eukaryotic cells and are a powerful tool for studying the origin of species, genetic diversity, and phylogenetics [4]. Mitochondrion and chloroplast are organelles with a semi-autonomous genetic system in higher plant cells, and they carry relevant genetic information. To date, 7367 chloroplast genomes and 1118 plastomes have been published in the NCBI database. However, only 441 plant mitochondrial genomes exist, according to the NCBI database (2022, April 23th, https://www.ncbi.nlm.nih.gov/genome/browse/#!/organelles/). Mitochondria play an important role in plant phylogeny and are widely used in phylogenetic and interspecies discrimination studies because their genetic system is relatively independent of the nucleus and relatively conserved [16]. In addition, studies have shown that mitochondria are closely related to cytoplasmic male sterility (CMS) and that self-incompatibility and hybrid incompatibility exist in sweet potato. Therefore, the study of the sweet potato mitochondrial genome can help identify and utilize wild seed resources of sweet potato, study the molecular mechanism of hybrid sterility, and provide services for sweet potato variety improvement [17, 18]. The study of these genomes has lagged behind that of chloroplast and plastid genomes because of the complex structure of plant mitochondrial genomes. Currently, 15 mitochondrial genomes have been published in the convolvulaceae family, while the mitochondrial genomes of sweet potato remain unreported.

In this research, we sequenced and assembled the mitochondrial genome of the sweet potato and further investigated its substructure. We explored the existence of its mitochondrial genomic substructure through nanopore reads and PCR experiments. To better study its mitochondrial and chloroplast genome homologous fragments, we assembled its chloroplast genome by using the same set of data and completed a sequence similarity analysis. We also analyzed its mitochondrial genome for RNA editing events. This study further demonstrates the existence of multiple conformations in plant mitochondrial genomes and provides a theoretical basis for the evolution of higher plants and CMS breeding.

## Material and methods

### Plant materials, DNA extraction, and sequencing

Fresh leaves of sweet potato plants (JinShan 57) were collected in Fujian Agriculture and Forestry University (Longitude: 119.306239, Latitude: 26.075302). The leaves were cleaned with DEPC water and stored in a freezer at − 80 °C. The DNA of sweet potato was extracted by using a DNA plant extraction kit (Tiangen, China). The short-paired reads were sequenced by Illumina HiSeq X ten (Illumina, Inc.; San Diego, CA, USA). For Oxford Nanopore sequencing, gTube was used to break genomic DNA into about 10 kb on average. The DNA library was taken, mixed with relevant reagents on board, and added to Flowcell for real-time single-molecule sequencing on the PromethION sequencer to obtain raw sequencing data.

### Genome assembly, polish, and annotation

For the chloroplast genome assembly, we used GetOrganelle [19] to assemble the whole genome sequencing (WGS) data with default parameters directly. Then, the chloroplast genome fragments generated from GetOrganelle were used to filter the raw nanopore and illumina data. For the mitochondrial genome assembly, we first used the Nextdenovo (https://github.com/Nextomics/NextDenovo) software de novo assembly with the nanopore reads and selected them with the reference genome *Ipomoea nil* (NC_031158.1). The mitochondrial genome results were manually checked by drawing the dotplot by Gepard [20]. The illumina data were assembled into a uniting graph by GetOrganelle. The graph-based mitochondrial genome was visualized by Bandage and used to remove the chloroplast- and nuclear-derived uniting nodes manually. The repeat regions were solved with the long-read results from NextDenovo.

The chloroplast genome was annotated with the CPGAVAS2 [21] and checked by CPGview-RSG (http://www.herbalgenomics.org/cpgview/,unpublished). The PCGs and rRNA of mitogenome were annotated with Geseq [22] and BLASTN. The tRNAs were identified with tRNAscan [23] (version 1.4). The genome circle map of mitochondrial genome was visualized with OGdraw [24]. All the annotations of organelle genomes were reviewed carefully and manually corrected with Apollo software [25, 26].

### Repeat and homologous DNA analysis

Microsatellite repeats were identified by MISA [27] with the parameters “1-10 2-6 3-5 4-5 5-5 6-5.” Dispersed repeats were found with REPuter web server (https://bibiserv.cebitec.uni-bielefeld.de/reputer/) with default parameters. Homologous DNA fragments were discovered between chloroplast genome and mitochondrial genome by BLASTN with the e-value of 1*e*-6 and word size 7 [28].

### Repeat-mediated homologous recombination prediction and PCR amplification validation

BLASTN was used to detect the paired repeat sequences. The potential recombination was identified from repeat sequences. Each pair of repeats and its neighboring 1000 bp were extracted as two template sequences. After that, we formed the other two conformations after recombination by exchanging the neighboring 1000 bp sequences. The long reads were mapped to the template sequences and carefully checked to see which one can pass the repeats for four template sequences. The primers at the ends of repeats were designed with IDT web server and shown in Table S1. The PCR amplification protocol was 50 μl in total, consisting of 2 μl Template DNA, 2.5 μl forward primer, 2.5 μl reverse primer, 25 μl 2x Unique™ Taq Super Master Mix, and 18 μl ddH_2_O. After initial denaturation at 94 °C for 3 min, PCR reactions were conducted for 35 cycles. Each cycle included denaturation at 94 °C for 10 s, annealing at 55 °C for 20 s, and elongation at 72 °C for 15 s. After the cycles ended, they were eventually extended for an additional 5 minutes.

### Phylogenetic analysis and RNA editing sites prediction

Sixteen mitochondrial genomes from convolvulaceae species were chosen for phylogenetic analysis, and *Asclepias syriaca* (Mitochondrion: NC_022796.1) was set as the outgroup. The common genes of mitochondrial genomes were filtered with BLASTN and extracted, concatenated with Phylosuite [29]. Multiple sequence alignment was conducted by using MAFFT [30]. The alignment results were calculated with MRBAYES [31]. Finally, the maximum-likelihood tree was visualized using iTOL (https://itol.embl.de/) [32]. We first downloaded the RNA-seq data from NCBI SRA database with the accession number DRR299538. We then decompressed this data using the SRAtoolkit [33] software to obtain the paired-end raw data. The protein-coding gene (PCG) sequences were extracted by using the Phylosuite software [29]. The RNA-seq data were mapped to all the PCGs of mitochondrial genome by using the BWA software [34]. The possible RNA editing sites were identified by using the Samtools program [35] according to the BAM file, and the locations with a coverage depth of more than 10x were selected.

## Results

### Mitochondrial genome assembly and annotation of sweet potato

**Fig. 1:**
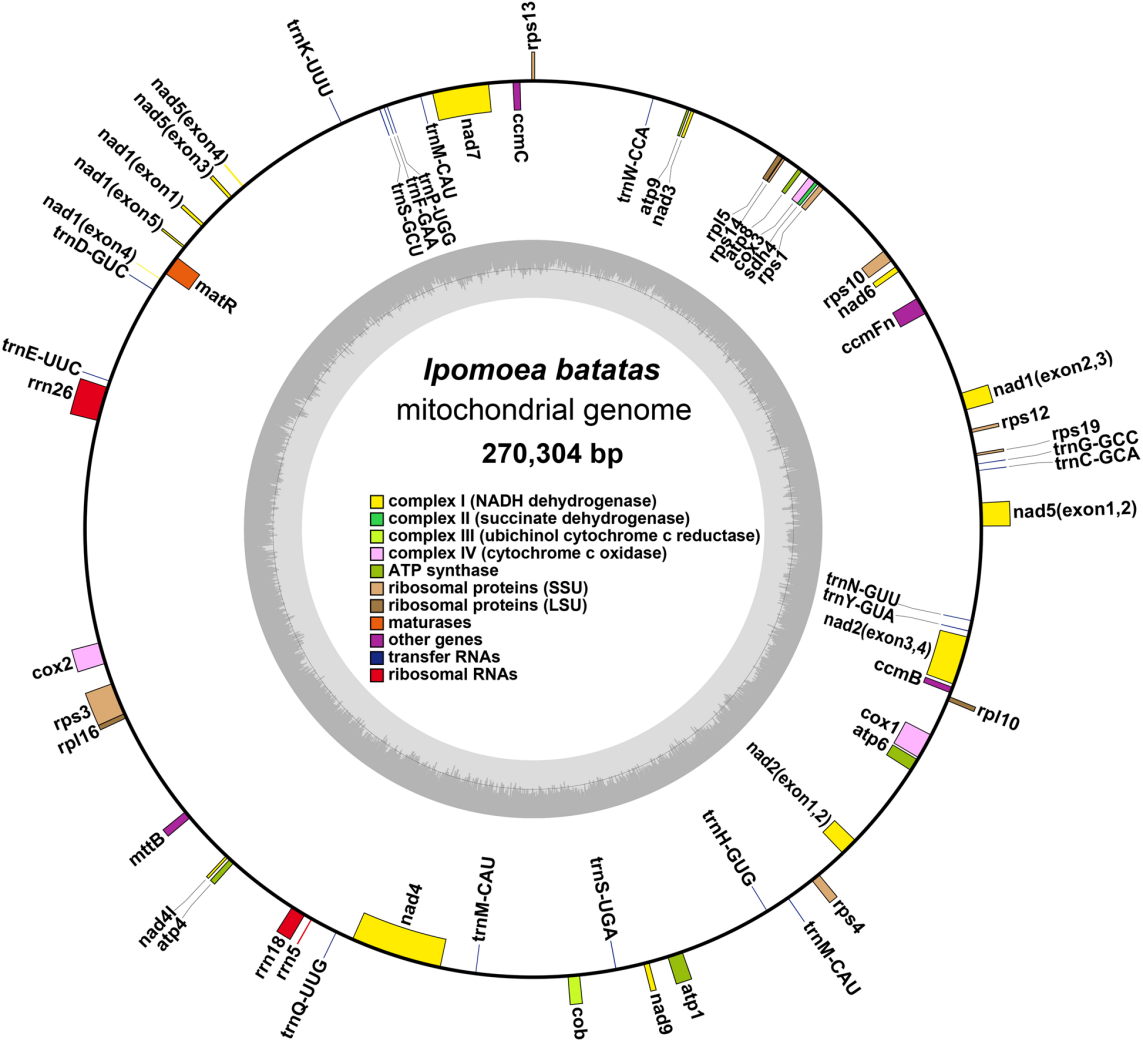
Representative genome map representing the mitochondrial genome circular molecule of *Ipomoea batatas*. The colored squares distributed inside and outside the circle represent different mitochondrial genes. Gene taxa of the same function are represented using the same color. In this case, the trans-shear genes nad1, nad2, nad5, etc. are represented as multiple exons

In total, 10 GB Illumina reads and 10 GB nanopore were generated for genome assembly (SRA number: SRR17210632, SRR17210633). The plant mitochondrial genome exhibits a complex conformation due to a large number of repeat sequences with NGS data (Figure S1) [36]. The nanopore raw reads were assembled into a circular molecular structure. The single circle was used to resolve the repeats on the graph. We adopted a hybrid assembly model and presented its mitochondrial genome temporarily in the form of a molecular circle with 270,304 bp (Fig. 1). In total, we filtered 2.8 Gb nanopore reads and 278 Mb Illumina reads for organelle. The coverage depth of mitochondrial genome is 312.97 x for long reads and 332.9 x for short reads. The GC contents of the mitochondrial genome were 44.08%.

We annotated the mitochondrial genome, and the categorization of genes is shown in Table 1. The sweet potato mitochondrial genome has 23 unique core genes and 12 variable genes. The core genes consist of five ATP synthase genes (*atp*1, *atp*4, *atp*6, *atp*8, and *atp*9), nine NADH dehydrogenase genes (*nad*1, *nad*2, *nad*3, *nad*4, *nad*4L, *nad*5, *nad*6, *nad*7, and *nad*9), three cytochrome C biogenesis genes (*ccm*B, *ccm*C, *ccm*Fn), three cytochrome C oxidase genes (*cox*1, *cox*2, and *cox*3), ubiquinol cytochrome c reductase (*co*b), a transport membrane protein (*mtt*B), and a maturase (*mat*R). The variable genes consist of three large subunits of ribosome proteins (*rpl*5, *rpl*10, and *rpl*16), eight small subunits of ribosome proteins (*rps*1, *rps*3, *rps*4, *rps*10, *rps*12, *rps*13, *rps*14, and *rps*19), and one respiratory gene (*sdh*4). *ccm*Fc lost some part of exons and became a pseudogene. In all, 6 rRNAs and 16 tRNA were annotated in the sweet potato mitochondrial genome (Table 1). Among the core genes, the *ccm*Fc gene has become a pseudogene due to the loss of part of the first exon. No PCGs are in duplicated regions, and all PCGs are single-copy.

**Table 1:** Gene composition in the mitogenome of sweet potato

Group of genes		Name of genes
Core genes	ATP synthase	*atp1*, *atp*4, *atp*6, *atp*8, *atp*9
	Cytochrome c biogenesis	*ccm*B, *ccm*C, *ccm*Fn, *ccm*Fc^a^
	Ubiquinol cytochrome c reductase	*cob*
	Cytochrome c oxidase	*cox1*, *cox*2, *cox*3
	Maturases	*mat*R
	Transport membrane protein	*mtt*B
	NADH dehydrogenase	*nad*1, *nad*2, *nad*3, *nad*4, *nad*4L, *nad*5, *nad*6, *nad7, nad*9
Variable genes	Large subunit of ribosome	*rpl*5, *rpl*10, *rpl*16
	Small subunit of ribosome	*rps*1*, rps*3, *rps*4, *rps*10, *rps*12, rps13, *rps*14, *rps*19
	Succinate dehydrogenase	*sdh*4^2^
rRNA genes	Ribosomal RNAs	*rrn*5 (×2), *rrn*8 (×2), *rrn*26 (× 2)^2^
tRNA genes	Transfer RNAs	*trn*C-GCA*, trn*D-GUC*, trn*E-UUC*, trn*F-GAA*, trn*G-GCC*, trn*H-GUG*, trn*K-UUU*, trn*M-CAU*, trn*M-CAU*, trn*M-CAU*, trn*N-GUU*, trn*P-UGG*, trn*Q-UUG*, trn*S-GCU*, trn*S-UGA*, trn*W-CCA*, trn*Y-GUA

^a^pseudogene

### Repeats mediate the homologous recombination

A single circular molecule is not sufficient to display the plant mitochondrial genome. Repeated sequences may be able to mediate varying degrees of rearrangement of the genome. To further investigate the possible occurrence of homologous recombination in the mitochondrial genome of sweet potato, we detected 279 pairs of repeat sequences in the sweet potato mitochondrial genome by using BLASTN with the e-value 1*e*-5. On the basis of the long reads, each pair of repeat sequences was carefully checked, and we found that three pairs of reads may support homologous recombination (Table 2). The length of those repeats ranges from 62 to 253 bp. All the pairs of repeats were direct repeats, and each set of direct repeats may cause the mitochondria genome to form into two separate circular molecules according to our preliminary judgment.

**Table 2:** Validation of the homologous recombination in the mitogenome of sweet potato

Repeat name	Repeat 1	Repeat 2	Repeat 3
Identities	94.46	97.43	93.54
Length	253	78	62
Position-1	124,865–125,108	240,244–240,321	117,130–117,191
Position-2	159,977–160,225	245,199–245,275	191,028–191,089
E-value	1.33E-103	1.14E-29	5.39E-18
Type	Direct	Direct	Direct

**Fig. 2:**
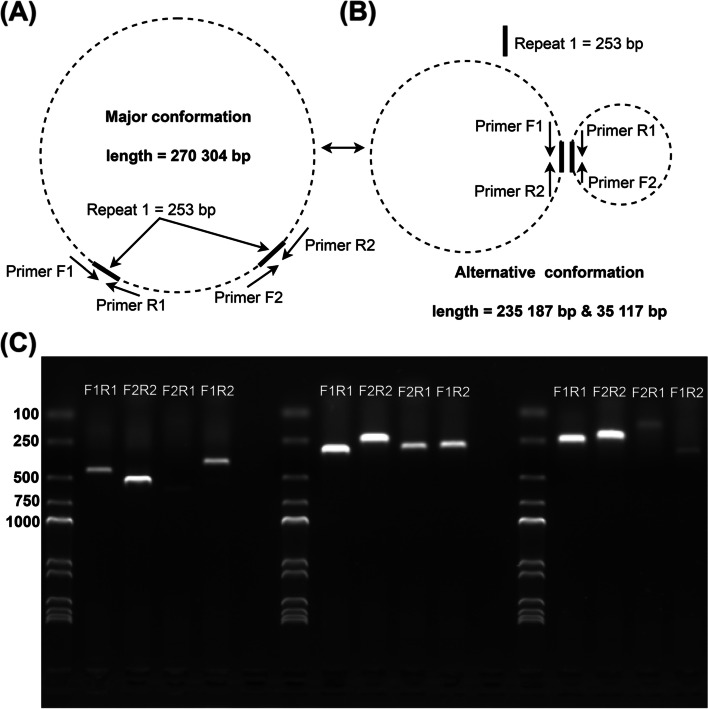
Validation of the homologous recombination mediated by different pair repeats. **A** Schematic of the primer design of direct repeats that could mediate the homologous recombination in the representative circular molecule. **B** Schematic of the experimental design following the exchange of reverse primers. **C** PCR production of validation results. From left to right, each of the five lanes represents a set of three experiments, namely, repeat1, repeat2, and repeat3. Each set of experiments is, from left to right, marker, major conformation 1, major conformation 2, alternative conformation 1, and alternative conformation 2

To further verify these potential homologous recombinations, we designed primers at each end of the repeat sequence for PCR amplification experiments (Table S1). Specific primers were designed on each side of the pair of direct repeats present on the main single circular molecule, allowing each of the two PCR products (primers F1 and R1, primers F2 and R2) to span the repeat sequence (Fig. 2A). When the conformation of the recombination was existent, the PCR product (primers F1 and R2, primers F2 and R1) of the exchange of reverse primers was also amplified (Figs. 2B and S2). As all three groups were positive replicates, we used the same strategy to design primers for the experiments, and the final results are displayed in Fig. 2C. All three sets of repeated sequences can produce secondary conformations, which is consistent with the results we obtained based on the long-read analysis.

**Fig. 3:**
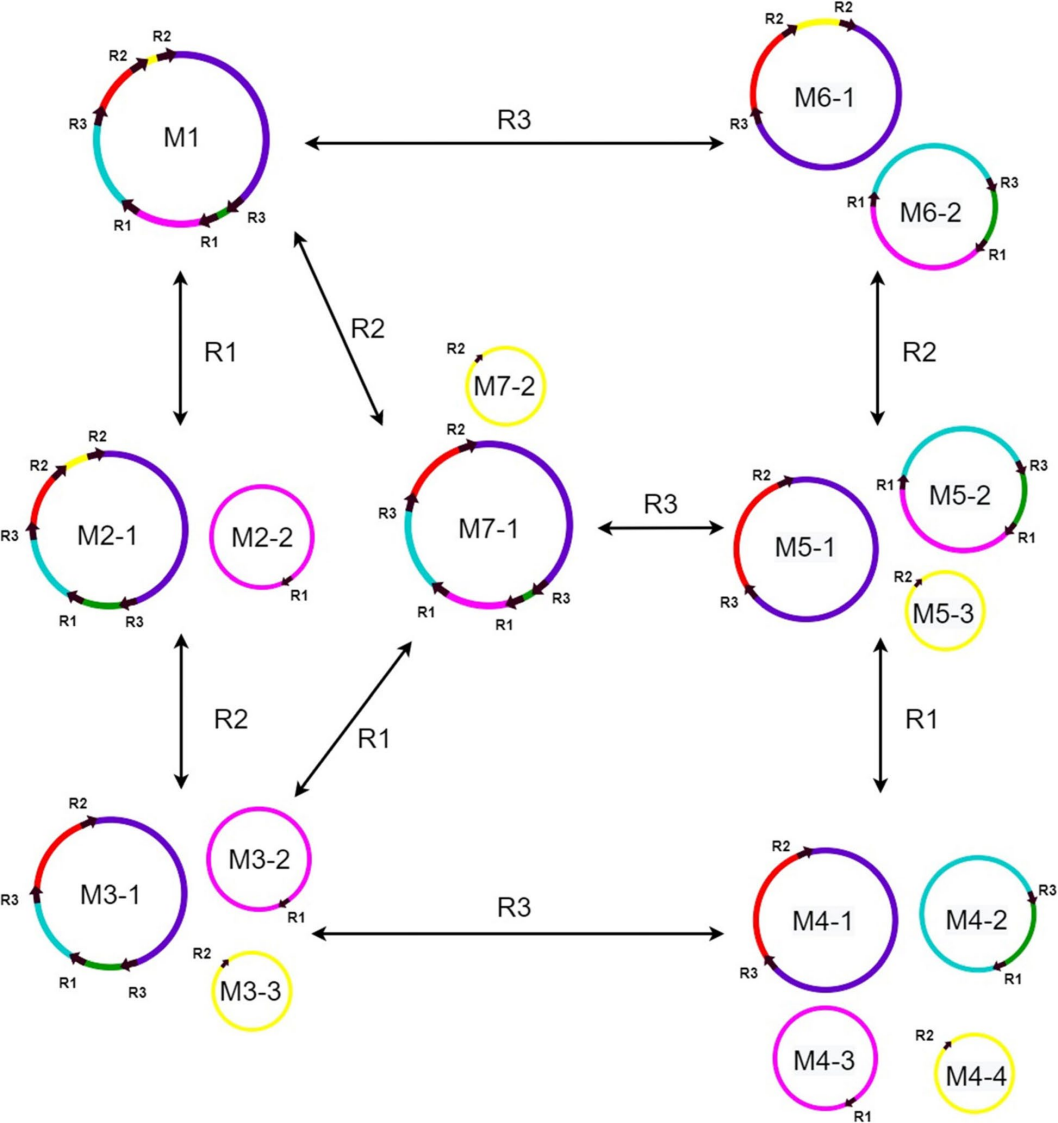
Hypothetical products generated by recombinations mediated by repeat1, repeat2, and repeat3. Repeats 1–3 were simply written as R1, R2, and R3 in the picture. The black arrows on the circular molecules represent the repeat sequences, and the colored lines represent the DNA fragment between the repeats. M1–M7 represent the conformations after rearrangement. M1 contains one circular molecule; M2, M6, and M7 contain two circular molecules; M3 and M5 contain three circular molecules; and M4 contains four circular molecules

On the basis of bioinformatics analysis and the results of validation by PCR experiments, we speculated on the potential forms of the mitochondrial genome (Fig. 3). M1 (only one molecule) was the representative structure, and it could form three minor conformations M2, M6, and M7 through the three direct repeats R1, R2, and R3, respectively, all of which were bicyclic. In addition, M2 could form three and four cyclic molecular structures through the two repeats of R2 and R3 (Fig. 3).

### Repeat elements analysis

**Fig. 4:**
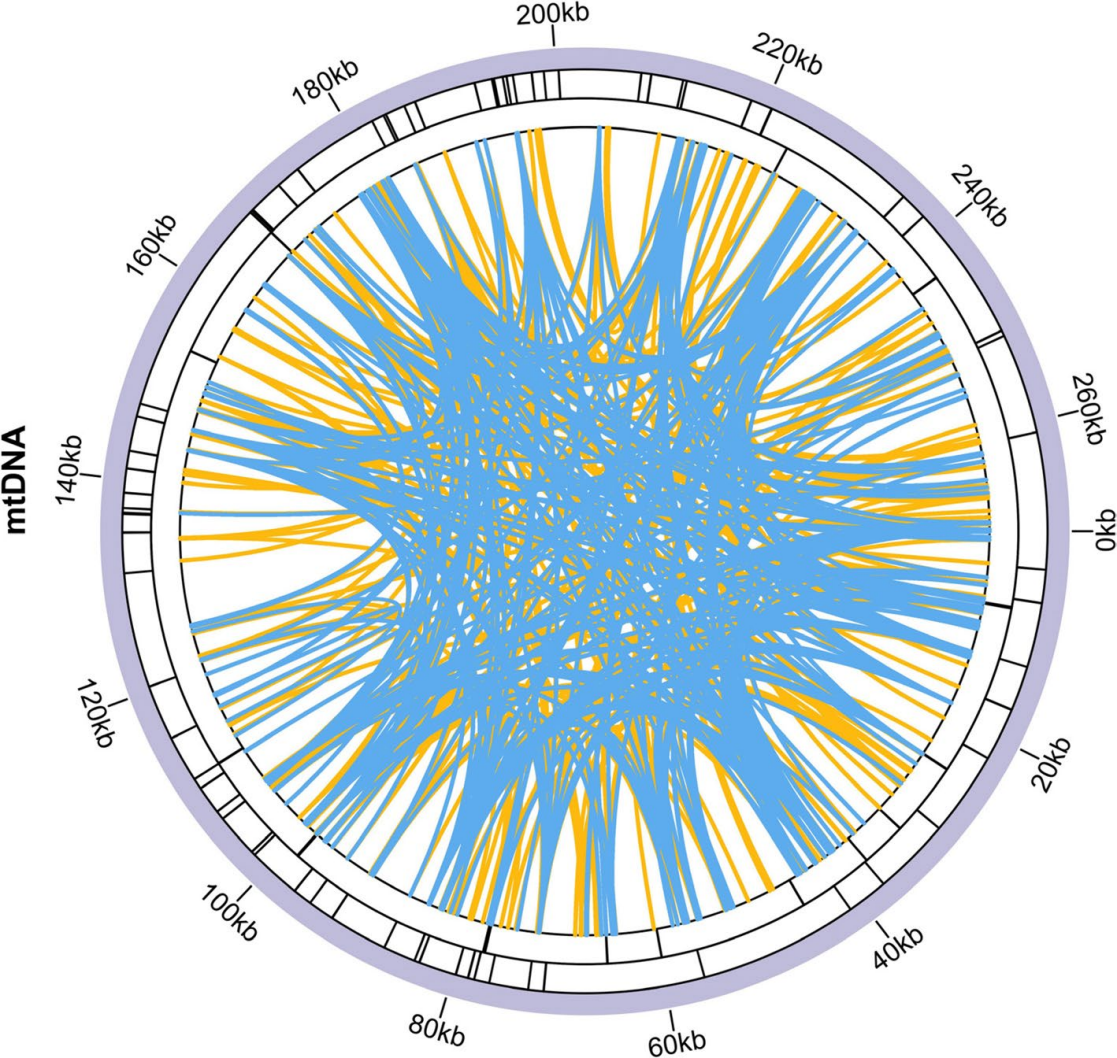
Repeat analysis of the mitochondrial genome in sweet potato*.* The inner circle shows the dispersed repeats connected with yellow and blue links. The next circles show the tandem repeats and microsatellite as short bars. The interval scale was 20 kb

Microsatellites (simple sequence repeats [SSRs]) are often used for molecular marker design because of their high polymorphism and codominant inheritance [37]. We used the Misa web server (https://webblast.ipk-gatersleben.de/misa/) to obtain the SSRs in the mitochondrial genome in sweet potato (Table S2, Fig. 4), and we identified 70 SSRs. In the mitochondrial genome, the most frequently occurring SSR was tetranucleotide, accounting for 41.4% SSRs (Table S2, Fig. 4). We also detected dispersed repeats in the mitochondrial genomes of sweet potato (Table S4–5). The dispersed repeats were drawn as colored lines linked at corresponding positions on the genome (Fig. 4). A total of 599 (forward: 303; palindromic: 296) dispersed repeats were found in the mitochondrial genome of sweet potato, and no complement repeats were found. The longest dispersed repeat in mitochondrial genome was palindromic repeat with a length of 915 bp (Table S3, Fig. 4). These repeat sequences have the potential to mediate genomic rearrangements and homologous recombination. In this study, we identified four sets of forward (direct) repeats that could form multiple circular molecules in the mitochondrial genome.

### DNA transfer and phylogenetic analysis

**Fig. 5:**
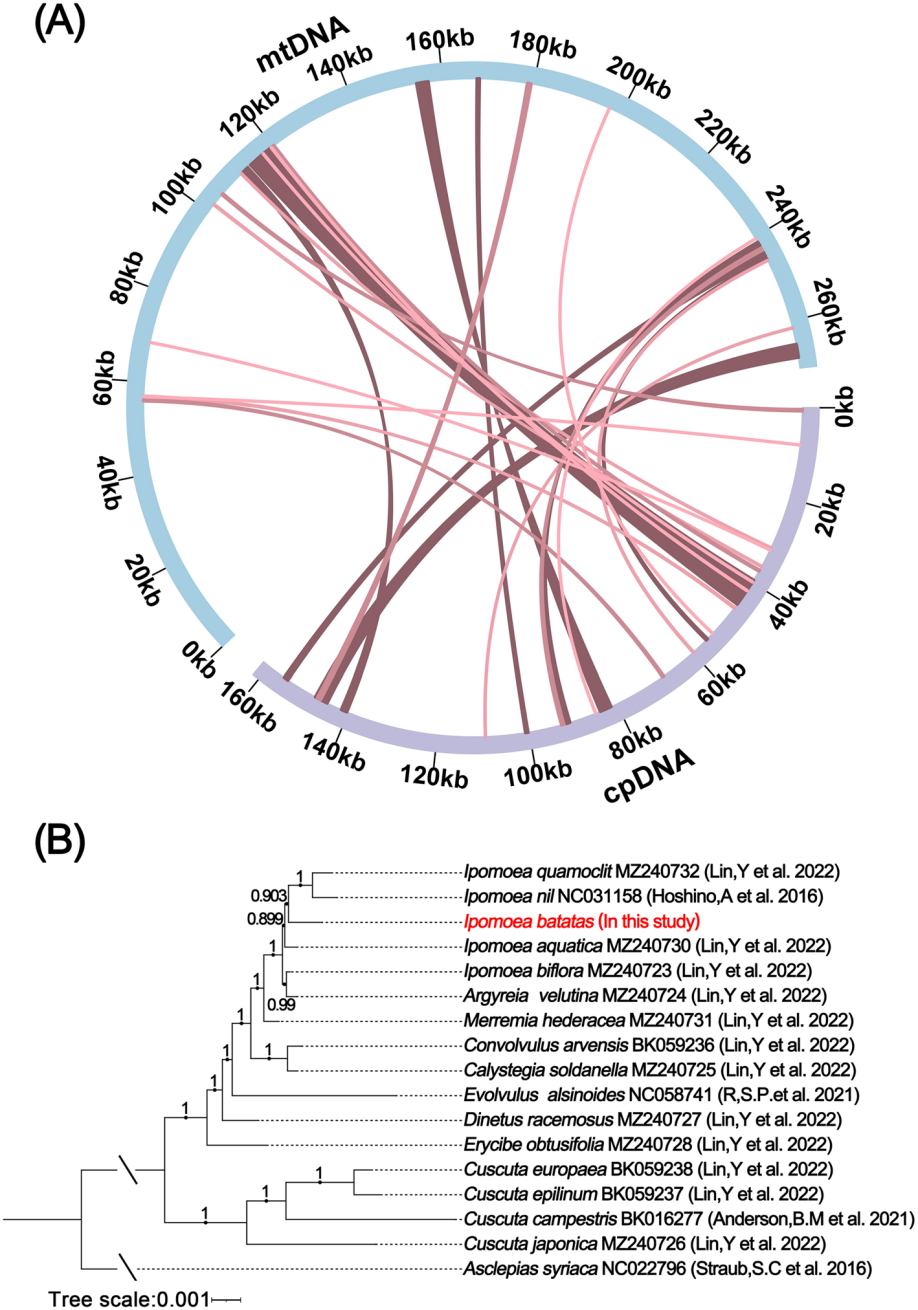
Homologous fragments and phylogenetic analysis of sweet potato*.* **A** DNA transfer between the chloroplast and mitochondrial genome. The blue circular segment represents the mitochondrial genome, and the purple circular segment represents the chloroplast genome. **B** Phylogenetic relationships of sweet potato

The chloroplast genome sequences were integrated into the mitochondrial genome during the evolution. We found 27 homologous DNA fragments in the sweet potato mitochondrial genome (Table S4, Fig. 5A). The summary insert length is 19,887 bp and accounts for 7.35% of the mitochondrial genome. To further explore the evolutionary relationships of mitochondria in sweet potato, we conducted a phylogenetic analysis of the mitochondria of 16 species of convolvulaceae (*Ipomoea quamoclit* MZ240732, *Ipomoea nil* NC031158, *Ipomoea batatas*, *Ipomoea aquatica* MZ240730, *Ipomoea biflora* MZ240723, *Argyreia velutina* MZ240724, *Merremia hederacea* MZ240731, *Convolvulus arvensis* BK059236, *Calystegia soldanella* MZ240725, *Evolvulus alsinoides* NC058741, *Dinetus racemosus* MZ240727, *Erycibe obtusifolia* MZ240728, *Cuscuta europaea* BK059238, *Cuscuta epilinum* BK059237, *Cuscuta campestris* BK016277, *Cuscuta japonica* MZ240726). These species have a large structural variation between them. Thus, we used a shared conserved PCG tree building approach. Twenty-four PCG (*atp*1, *atp*4, *atp*8, *ccm*C, *co*b, *cox*1, *cox*2, *mat*R, *nad*1, *nad*2, *nad*3, *nad*4, *nad*4L, *nad*5, *nad*6, *nad*7, *nad*9, *rpl*5, *rpl*16, *rps*3, *rps*4, *rps*12, *rps*13, and *rps*19) were used for phylogenetic analysis (Fig. 5B). All bootstraps exceeded 0.89, indicating the reliability of the inferences from the phylogenetic analysis. According to our analysis, four cuscuta species clustered into one clade and others clustered into one clade. The sweet potato (*I. batatas*) was closely related to the *I. nil* and *I. quamoclit*.

### RNA editing events in sweet potato

**Fig. 6:**
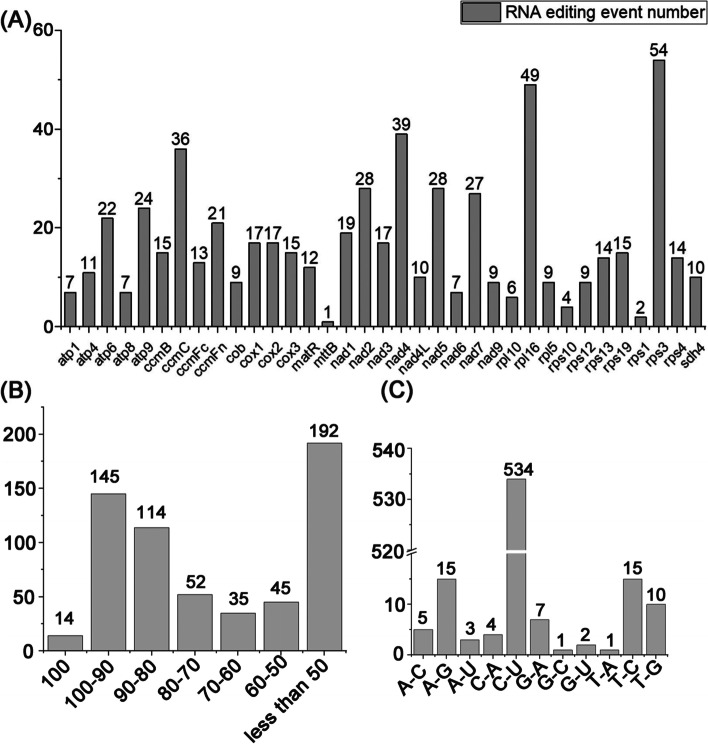
Prediction of RNA editing events in sweet potato*.* **A** Number of RNA editing sites in all the PCGs. **B** RNA editing efficiency in mitochondrial genome. **C** RNA editing type and their number identified in the mitochondrial genome

We predicted RNA editing events for 35 PCGs. A total of 597 RNA editing sites met the requirement of coverage depth > 10 and were distributed among all PCGs (Table S5). *ccm*C, *nad*4, *rpl*16 and *rps*3 genes were edited more than 30 times, and the *rps*3 gene was edited 54 times, which occurred most frequently (Fig. 6A). Fourteen sites had 100% editing efficiency, and 67.8% of the sites had more than 50% editing efficiency (Fig. 6B). We finally found 11 types of RNA editing in this mitochondrial genome and did not find C to G editing (Fig. 6C). C to U editing was present in all PCGs and has the highest number of occurrences (534 times, 89.4%). G to C and T to A editing were found in the *cox*2 and the *atp*9 gene only once. In summary, RNA editing events occur mainly on C to U and with appreciable efficiency.

## Discussion

In this study, the mitochondrial genomes of sweet potato were assembled, and potential substructures were identified. In assembling the mitochondrial genome, we used a graph-based genome that combined the assembly of NGS reads with long reads to assemble the mitochondrial genome of sweet potato (Figure S1). This approach has the advantage of avoiding inconsistencies in the use of NGS data to polish the results of the long-read assembly [38]. Long-read sequencing such as nanopore has a high sequencing error rate and by way of embellishment can lead to assembly results with incorrect bases, especially in protein-coding regions, which can further cause other problems such as stop codons in regions in the middle of PCGs in the mitochondrial genome [39].

Unlike the conserved monocyclic structure of plant chloroplast genomes, seed plant mitochondrial genomes generally have multiple alternative conceptions or minor conformations due to repeat sequences [40, 41]. Jingling Li found a 175 bp direct repeat that can divide the *Scutellaria tsinyunensis* mitochondrial genome into two dependent circular molecules [36]. Shanshan Dong reported that the mitochondrial genome of the *Nymphaea coloratal* may undergo low-frequency recombination under the influence of repeat sequences [28]. Shuaibin Wang discovered the existence of two circular molecules that differed greatly in size in the kiwi fruit mitochondrial genome [42]. The mitochondrial genomes of *Lactuca* were detected with a variety of linear, branched, and circular structures [43]. In summary, plant mitochondria are polymorphic and cannot be represented using only a single circular molecule. Three pairs of repeat sequences found in this study could allow the sweet potato mitochondrial genome to form four separate circular molecules. These phenomena may be due to the specific DNA repair mechanism in the plant mitochondrial genome [44]. We verified only that these cyclic structures existed, but whether those four circular molecules could exist simultaneously requires further investigation.

In addition, the RNA editing events in sweet potato mitochondrial genome were investigated. We found 597 RNA editing sites, which is highly similar to other land plants [45–50]. RNA editing events may affect the start or end points of PCGs. We hypothesized that the *rps*10 gene with start codon ACG appeared because a C to U editing event occurred at the starting position. However, we did not find transcriptome comparison reads to support this phenomenon. According to a previous report, the *cox*1 gene was transcribed through an RNA editing event that allowed ACG to be edited to AUG and was thus used as a starting point in potato [51]. However, ACG could be used directly as starting points for transcription without editing in organelle genomes [52]. Therefore, whether the starting point of rps10 in sweet potato is edited or not still needs further experimental verification.

Previous studies found that the mitochondrial genomes of the crops were associated with CMS and that the stability of the mitochondrial genome was regulated by nuclear gene expression [53–55]. The sweet potato nuclear genome is homozygous and heterozygous for six ploidy, and varieties can be divided into 15 infertile clusters [13]. Generally, self-crosses of the same variety are incompatible (self-fertility), crosses between varieties within a cluster are incompatible, and crosses between varieties in infertile clusters can bear fruit. The plant trait CMS is determined by the mitochondrial genome and is associated with a pollen sterility phenotype that can be suppressed or counteracted by nuclear genes known as restorer-of-fertility genes [54]. Dissecting the mitochondrial genome of sweet potato can provide a theoretical basis for CMS breeding in sweet potato.

## Conclusion

We successfully assembled the mitochondrial genome of sweet potato by using both nanopore reads and Illumina reads. The mitochondrial genome of sweet potato contains three pairs of direct repeats (253 bp, 78 bp, and 62 bp) which can mediate homologous recombination, and the mitochondrial genome of sweet potato could form a polycyclic structure under the influence of these repeats. Further studies may be conducted in the future to determine whether these circular molecules co-exist and how they relate to CMS breeding.

## Supplementary Information

**Additional file 1: Table S1.** The primers design for repeats mediate the homologous recombination *I. batatas.* **Table S2.** Microsatellite repeats in the mitogenome*.* **Table S3.** Dispersed repeats in the mitogenome. **Table S4.** The DNA transfer in the mitochondrial genome of *I. batatas****.*** **Table S5.** The RNA editing events prediction in the *I. batatas.* **Figure S1.** The graph-based mitochondrial genome of *I. batatas. ***Figure S2.**The raw Gel diagram of agarose gel electrophoresis.

## Data Availability

The mitogenome sequences supporting the conclusions of this article are available in GenBank (https://www.ncbi.nlm.nih.gov/) with accession numbers: OL699988. The mitochondrial genome also could download from the Figshare platform with the public links (https://figshare.com/s/7c78c9f9ae924d2281ec). The sample has been deposited in the Fujian Agriculture and Forestry University (Fuzhou, China) with accession number IP01. The raw data have been submitted to the SRA database (NGS: SRR17210632; ONT: SRR17210633).
