# Supplementary material for: De novo assembly of the complete mitochondrial genome of sweet potato (Ipomoea batatas [L.] Lam) revealed the existence of homologous conformations generated by the repeat-mediated recombination
Source: BMC Plant Biol. 2022 Jun 10;22:285. doi: 10.1186/s12870-022-03665-y (PMC9185937; doi:10.1186/s12870-022-03665-y)
Supplement: Supplementary file 1 — Additional file 1: Table S1. The primers design for repeats mediate the homologous recombination I. batatas. Table S2. Microsatellite repeats in the mitogenome. Table S3. Dispersed repeats in the mitogenome. Table S4. The DNA transfer in the mitochondrial genome of I. batatas. Table S5. The RNA editing events prediction in the I. batatas. Figure S1. The graph-based mitochondrial genome of I. batatas. Figure S2.The raw Gel diagram of agarose gel electrophoresis. [file 12870_2022_3665_MOESM1_ESM.pdf]

Table S1 The primers design for repeats mediate the homologous recombination *I. batatas.*

Repeat number	Primer name	Primer sequence
Repeat1	F1	CTATCCCATCCCGTTCCTTTATC
	R1	GTATCATCCACACCCAGTCAC
	F2	GTCCAGCTTACGGACATAACA
	R2	TAGGAACCTCCAAACCAAGAAG
Repeat2	F1	CGCATAGATTCCGCCTAAGAG
	R1	CCTTGAACTACCGGAGCATTAT
	F2	GTGGGAGTTAGAGCAAGAGTTAG
	R2	CCTGTGGCTGGCTATCTTATC
Repeat3	F1	CTTCACGCCCTTTCATTCTTATTC
	R1	ATTCGAGGCGTCAGCAAA
	F2	TACTCGGACTTGGGTCTCTC
	R2	GGGTTGCTCTGGGATAAAGT

Table S2 Microsatellite repeats in the mitogenome. The structure of Microsatellite repeats is presented as repeat units surrounded with parenthesis and the numbers of repeat units.

Type	Structure of Microsatellite Repeats	Size (bp)	Start	End
p1	(T)11	11	3590	3600
p4	(AAGA)3	12	6730	6741
p4	(TAAA)3	12	12835	12846
p6	(GGCCAA)3	18	17053	17070
p6	(GGTCCT)3	18	22083	22100
p4	(AAGA)3	12	30048	30059
p3	(GAA)4	12	37363	37374
p4	(AATT)3	12	41255	41266
p4	(CTTA)3	12	56310	56321
p4	(AATA)3	12	71407	71418
p2	(TA)5	10	72849	72858
p2	(TA)5	10	76540	76549
p1	(A)10	10	77977	77986
p4	(TCTT)3	12	78565	78576
p4	(AAAG)3	12	79747	79758
p5	(ATTAC)3	15	83321	83335
p4	(CTTT)3	12	83803	83814
p3	(TAA)4	12	86863	86874
p4	(AAGG)3	12	92437	92448
p4	(GTGA)3	12	94926	94937
p6	(GACAGG)3	18	96744	96761

p2	(CT)5	10	101754	101763
p2	(AT)5	10	102138	102147
p4	(TTCA)3	12	106239	106250
p5	(ACTAG)3	15	107082	107096
p1	(A)10	10	109742	109751
p2	(AT)5	10	110782	110791
p2	(TA)6	12	115146	115157
p1	(T)10	10	120392	120401
p4	(AAAG)3	12	131210	131221
p1	(A)10	10	135025	135034
p3	(TAA)4	12	136768	136779
p4	(AAGA)3	12	136864	136875
p3	(TAA)4	12	137314	137325
p2	(GA)5	10	138761	138770
p4	(AAAG)3	12	141026	141037
p4	(AAGC)3	12	142693	142704
p4	(GAGC)3	12	145975	145986
p1	(T)10	10	147206	147215
p5	(AATAG)4	20	167955	167974
p2	(CT)5	10	168178	168187
p4	(ACCT)3	12	168283	168294
p3	(TCT)4	12	171601	171612
p3	(TCT)4	12	173891	173902
p4	(ACCG)3	12	182160	182171
p2	(AG)5	10	183347	183356

p1	(T)11	11	183597	183607
p4	(ACAA)3	12	185476	185487
p4	(TAAA)3	12	186528	186539
p2	(AG)6	12	192358	192369
p1	(T)10	10	193998	194007
p4	(TTTC)3	12	194190	194201
p4	(CTGG)3	12	195039	195050
p1	(A)10	10	195396	195405
p4	(TTCT)3	12	195990	196001
p4	(GAAT)3	12	197763	197774
p4	(CCTT)3	12	198897	198908
p4	(AGAA)3	12	200282	200293
p2	(AT)5	10	208006	208015
p1	(A)10	10	208935	208944
p2	(CT)5	10	211947	211956
p4	(ACTT)3	12	212290	212301
p1	(T)10	10	218580	218589
p3	(TTC)4	12	220589	220600
p6	(CTTATT)3	18	220618	220635
p2	(AT)9	18	235423	235440
p2	(AG)5	10	238040	238049
p1	(T)10	10	250971	250980
p3	(TTC)4	12	251492	251503
p4	(TGAA)3	12	260990	261001

Table S3 Dispersed repeats in the mitogenome.

The repeat length of the first part	The starting site of the first part	Matching direction	The repeat length of the second part	The starting site of the second part	interval distance of repeats	E-value
915	8351	P	915	51703	0	0.00E+00
562	58162	F	562	218675	0	0.00E+00
382	10137	F	382	215700	0	2.12E-220
289	10512	F	289	163556	-3	2.23E-156
264	64325	F	264	213144	0	2.34E-149
189	106440	F	189	177381	0	3.34E-104
193	40238	P	193	163451	-1	7.55E-104
170	212786	P	170	261907	0	9.17E-93
163	121161	F	163	227243	0	1.50E-88
142	29011	F	142	77686	0	6.61E-76
140	124864	F	140	159976	0	1.06E-74
137	65820	P	137	74780	0	6.77E-73
145	105132	P	145	269458	-2	9.71E-73
129	171859	P	129	261961	0	4.44E-68
128	9723	P	128	42677	0	1.77E-67

126	64429	P	126	249836	0	2.84E-66
126	213248	P	126	249836	0	2.84E-66
121	56632	P	121	84571	0	2.91E-63
124	83106	F	124	84570	-1	1.69E-62
130	28986	F	130	77556	-3	1.07E-61
118	146002	P	118	167429	0	1.86E-61
121	56632	P	121	83107	-1	1.06E-60
116	171872	F	116	212786	0	2.98E-60
117	41473	P	117	84563	-1	2.61E-58
112	25072	P	112	83107	0	7.62E-58
111	36411	F	111	105196	0	3.05E-57
110	41473	P	110	83106	0	1.22E-56
109	25075	F	109	41473	0	4.88E-56
112	25072	F	112	56641	-1	2.56E-55
112	25072	P	112	84571	-1	2.56E-55
105	29011	F	105	77581	0	1.25E-53
105	77581	F	105	77686	0	1.25E-53
105	184097	P	105	270106	0	1.25E-53
109	41473	F	109	56644	-1	1.60E-53
104	26391	P	104	171359	0	5.00E-53
103	39330	P	103	106442	0	2.00E-52
103	39330	P	103	177383	0	2.00E-52
103	18834	F	103	72462	-1	6.17E-50
96	106441	P	96	232725	0	3.27E-48
96	177382	P	96	232725	0	3.27E-48
95	39338	F	95	232725	0	1.31E-47
92	55093	F	92	255547	0	8.38E-46

92	146029	F	92	270211	0	8.38E-46
91	167429	P	91	270211	0	3.35E-45
90	72415	F	90	214325	0	1.34E-44
90	98702	F	90	214369	0	1.34E-44
89	98704	F	89	270215	0	5.36E-44
88	10512	P	88	40238	0	2.15E-43
88	41502	P	88	84563	0	2.15E-43
88	98704	F	88	146033	0	2.15E-43
88	146033	F	88	214371	0	2.15E-43
88	214371	F	88	270215	0	2.15E-43
98	8706	F	98	228602	-3	8.40E-43
98	52165	P	98	228602	-3	8.40E-43
87	41503	F	87	72485	0	8.58E-43
87	72485	P	87	84563	0	8.58E-43
87	98704	P	87	167429	0	8.58E-43
87	167429	P	87	214371	0	8.58E-43
85	148316	P	85	169845	0	1.37E-41
82	10244	F	82	269341	0	8.79E-40
82	150082	P	82	254874	0	8.79E-40
82	215807	F	82	269341	0	8.79E-40
89	5743	P	89	41384	-2	1.89E-39
85	72480	P	85	195418	-1	3.50E-39
81	18856	F	81	72484	0	3.52E-39
81	18857	P	81	83105	0	3.52E-39
80	18857	F	80	41503	0	1.41E-38
80	18857	P	80	84570	0	1.41E-38
80	25104	F	80	56673	0	1.41E-38

80	25104	P	80	84571	0	1.41E-38
80	41502	F	80	56673	0	1.41E-38
80	56673	P	80	83107	0	1.41E-38
80	72485	P	80	83106	0	1.41E-38
87	52176	P	87	228602	-2	2.89E-38
79	18857	F	79	25105	0	5.62E-38
79	18857	F	79	56674	0	5.62E-38
79	18858	P	79	195418	0	5.62E-38
79	25105	F	79	72485	0	5.62E-38
79	41504	P	79	195418	0	5.62E-38
79	56674	F	79	72485	0	5.62E-38
79	83106	F	79	195418	0	5.62E-38
79	84570	F	79	195418	0	5.62E-38
82	8722	F	82	228618	-1	2.16E-37
78	25106	P	78	195419	0	2.25E-37
78	56675	P	78	195419	0	2.25E-37
81	586	F	81	229300	-1	8.54E-37
75	39326	P	75	214395	0	1.44E-35
75	83166	F	75	232768	0	1.44E-35
85	36411	P	85	269454	-3	3.66E-35
78	180864	F	78	214394	-1	5.26E-35
73	125035	F	73	160152	0	2.30E-34
82	7272	F	82	150208	-3	2.10E-33
71	39330	P	71	180865	0	3.69E-33
71	106474	F	71	180865	0	3.69E-33
71	106474	F	71	214395	0	3.69E-33
71	177415	F	71	180865	0	3.69E-33

71	177415	F	71	214395	0	3.69E-33
74	240247	F	74	245201	-1	1.28E-32
70	195538	F	70	232779	0	1.47E-32
73	8121	P	73	38243	-1	5.05E-32
72	13463	F	72	232716	-1	1.99E-31
68	13468	P	68	180864	0	2.36E-31
68	13468	P	68	214394	0	2.36E-31
67	13468	F	67	39334	0	9.44E-31
67	13468	P	67	106474	0	9.44E-31
67	13468	P	67	177415	0	9.44E-31
66	13392	F	66	84505	0	3.77E-30
65	13471	P	65	98727	0	1.51E-29
65	13471	P	65	146056	0	1.51E-29
65	13471	P	65	270238	0	1.51E-29
65	98727	F	65	180864	0	1.51E-29
65	146056	F	65	180864	0	1.51E-29
65	167427	F	65	232723	0	1.51E-29
65	180864	F	65	270238	0	1.51E-29
68	82131	P	68	180313	-1	4.81E-29
68	148268	P	68	204297	-1	4.81E-29
64	13472	F	64	167429	0	6.04E-29
64	39337	P	64	98728	0	6.04E-29
64	39337	P	64	146057	0	6.04E-29
64	39337	P	64	270239	0	6.04E-29
64	43831	P	64	51382	0	6.04E-29
64	83177	F	64	195538	0	6.04E-29
64	98728	F	64	106474	0	6.04E-29

64	98728	F	64	177415	0	6.04E-29
64	106474	F	64	146057	0	6.04E-29
64	106474	F	64	270239	0	6.04E-29
64	146057	F	64	177415	0	6.04E-29
64	167429	P	64	180864	0	6.04E-29
64	177415	F	64	270239	0	6.04E-29
71	7283	F	71	150219	-2	8.24E-29
63	13376	F	63	56463	0	2.42E-28
63	13472	F	63	232725	0	2.42E-28
63	39338	F	63	167429	0	2.42E-28
63	98728	P	63	232725	0	2.42E-28
63	106474	P	63	167429	0	2.42E-28
63	146057	P	63	232725	0	2.42E-28
63	167429	P	63	177415	0	2.42E-28
63	180865	P	63	232725	0	2.42E-28
63	214395	P	63	232725	0	2.42E-28
63	232725	P	63	270239	0	2.42E-28
62	7563	F	62	7647	0	9.66E-28
61	72515	P	61	143806	0	3.86E-27
64	84630	F	64	232768	-1	1.16E-26
60	4929	F	60	106556	0	1.55E-26
60	4929	F	60	177497	0	1.55E-26
60	39419	P	60	214323	0	1.55E-26
67	228245	P	67	248268	-2	1.88E-26
62	56632	P	62	232768	-1	1.80E-25
58	7296	F	58	150232	0	2.47E-25
58	39419	P	58	72415	0	2.47E-25

68	179180	F	68	266188	-3	3.19E-25
57	41533	P	57	143810	0	9.89E-25
57	83008	P	57	106572	0	9.89E-25
57	83008	P	57	177513	0	9.89E-25
57	84563	F	57	143810	0	9.89E-25
57	100274	P	57	253284	0	9.89E-25
56	106381	P	56	143872	0	3.96E-24
55	25070	F	55	106439	0	1.58E-23
62	891	F	62	150835	-2	1.64E-23
65	39061	P	65	215613	-3	1.78E-23
65	178600	F	65	265605	-3	1.78E-23
65	227943	P	65	248563	-3	1.78E-23
54	25071	F	54	177381	0	6.33E-23
57	8768	F	57	180165	-1	1.69E-22
57	52144	P	57	180165	-1	1.69E-22
53	25072	P	53	232768	0	2.53E-22
53	83166	P	53	106441	0	2.53E-22
53	83166	P	53	177382	0	2.53E-22
56	67370	F	56	227533	-1	6.65E-22
62	56610	P	62	232790	-3	9.87E-22
52	25073	P	52	39381	0	1.01E-21
52	39381	F	52	83166	0	1.01E-21
55	41571	F	55	55413	-1	2.61E-21
60	114115	P	60	251355	-3	1.43E-20
50	18887	P	50	143817	0	1.62E-20
50	39381	P	50	41473	0	1.62E-20
50	41473	F	50	106444	0	1.62E-20

50	41473	F	50	177385	0	1.62E-20
50	41473	P	50	232768	0	1.62E-20
50	83106	F	50	143817	0	1.62E-20
50	143817	F	50	195418	0	1.62E-20
53	56641	F	53	106441	-1	4.03E-20
53	56641	F	53	177382	-1	4.03E-20
53	84630	P	53	106441	-1	4.03E-20
53	84630	P	53	177382	-1	4.03E-20
53	84641	F	53	195538	-1	4.03E-20
59	56613	P	59	195549	-3	5.43E-20
56	227952	P	56	248563	-2	5.49E-20
49	7215	F	49	150156	0	6.48E-20
49	12997	P	49	235903	0	6.48E-20
49	25075	F	49	234243	0	6.48E-20
49	25135	P	49	143818	0	6.48E-20
49	39382	P	49	234243	0	6.48E-20
49	41473	F	49	234243	0	6.48E-20
49	56704	P	49	143818	0	6.48E-20
49	83167	P	49	234243	0	6.48E-20
49	106444	F	49	234243	0	6.48E-20
49	177385	F	49	234243	0	6.48E-20
49	232769	P	49	234243	0	6.48E-20
52	39381	P	52	56642	-1	1.58E-19
52	39381	F	52	84630	-1	1.58E-19
51	56632	P	51	195538	-1	6.20E-19
57	97392	F	57	180841	-3	7.82E-19
47	56479	F	47	84505	0	1.04E-18

50	36442	P	50	269458	-1	2.43E-18
50	64644	P	50	100142	-1	2.43E-18
50	105227	P	50	269458	-1	2.43E-18
56	100341	P	56	121266	-3	2.96E-18
56	100341	P	56	227348	-3	2.96E-18
53	39663	F	53	234459	-2	3.14E-18
46	72459	F	46	98702	0	4.15E-18
46	84537	P	46	106617	0	4.15E-18
49	56644	F	49	234243	-1	9.53E-18
49	84631	P	49	234243	-1	9.53E-18
49	117129	F	49	191027	-1	9.53E-18
52	13514	P	52	106383	-2	1.21E-17
52	13514	F	52	143874	-2	1.21E-17
51	39075	P	51	215613	-2	4.65E-17
44	4945	P	44	83021	0	6.64E-17
44	72461	F	44	146033	0	6.64E-17
44	72461	P	44	167472	0	6.64E-17
44	72461	F	44	270215	0	6.64E-17
44	212935	P	44	214300	0	6.64E-17
47	39192	P	47	215534	-1	1.46E-16
53	67453	F	53	227616	-3	1.60E-16
53	110376	P	53	262122	-3	1.60E-16
52	137089	F	52	250904	-3	6.05E-16
42	25072	P	42	195538	0	1.06E-15
42	83188	F	42	84652	0	1.06E-15
42	84652	F	42	195549	0	1.06E-15
42	84652	F	42	232790	0	1.06E-15

42	96557	F	42	106734	0	1.06E-15
42	106441	P	42	195538	0	1.06E-15
42	177382	P	42	195538	0	1.06E-15
45	56936	P	45	81353	-1	2.24E-15
51	64264	P	51	83275	-3	2.28E-15
48	43530	P	48	253169	-2	2.63E-15
41	39392	F	41	195538	0	4.25E-15
41	215629	F	41	262848	0	4.25E-15
50	8654	F	50	9712	-3	8.58E-15
50	9712	P	50	52265	-3	8.58E-15
47	117331	F	47	119555	-2	1.01E-14
47	177734	P	47	191037	-2	1.01E-14
43	18834	F	43	98705	-1	3.43E-14
43	18834	F	43	146034	-1	3.43E-14
43	18834	P	43	167472	-1	3.43E-14
43	18834	F	43	214372	-1	3.43E-14
43	18834	F	43	270216	-1	3.43E-14
43	94539	P	43	180841	-1	3.43E-14
39	41473	P	39	195538	0	6.80E-14
39	55203	F	39	255677	0	6.80E-14
39	64655	P	39	100142	0	6.80E-14
39	110390	P	39	262122	0	6.80E-14
39	195538	P	39	234243	0	6.80E-14
48	91261	P	48	184136	-3	1.21E-13
48	91261	F	48	270124	-3	1.21E-13
45	77971	P	45	204285	-2	1.48E-13
47	72459	F	47	98641	-3	4.54E-13

47	227802	P	47	248716	-3	4.54E-13
41	65290	F	41	65302	-1	5.23E-13
44	18834	F	44	98644	-2	5.65E-13
46	98641	F	46	98702	-3	1.70E-12
46	98641	F	46	214369	-3	1.70E-12
46	103299	F	46	163354	-3	1.70E-12
40	39086	P	40	215613	-1	2.04E-12
36	44	F	36	8666	0	4.35E-12
36	44	P	36	52267	0	4.35E-12
36	180950	F	36	262093	0	4.35E-12
45	97637	P	45	269442	-3	6.36E-12
45	112367	P	45	124371	-3	6.36E-12
39	8665	P	39	42766	-1	7.96E-12
39	42766	F	39	52265	-1	7.96E-12
39	86805	F	39	151438	-1	7.96E-12
39	115911	P	39	115911	-1	7.96E-12
42	43339	F	42	148294	-2	8.23E-12
42	43339	P	42	204297	-2	8.23E-12
35	41564	F	35	106519	0	1.74E-11
35	41564	F	35	177460	0	1.74E-11
44	41441	F	44	270183	-3	2.37E-11
44	67434	F	44	227597	-3	2.37E-11
44	98643	F	44	146033	-3	2.37E-11
44	98643	P	44	167472	-3	2.37E-11
44	98643	F	44	270215	-3	2.37E-11
38	5718	P	38	13663	-1	3.10E-11
38	34804	P	38	178281	-1	3.10E-11

38	57592	P	38	83217	-1	3.10E-11
38	117139	P	38	177743	-1	3.10E-11
38	215269	P	38	239920	-1	3.10E-11
41	39069	P	41	262848	-2	3.14E-11
41	106118	F	41	191963	-2	3.14E-11
41	148276	P	41	264814	-2	3.14E-11
41	204316	F	41	264814	-2	3.14E-11
34	0	F	34	98793	0	6.96E-11
34	13424	P	34	106629	0	6.96E-11
34	227974	P	34	248563	0	6.96E-11
43	228043	P	43	248485	-3	8.85E-11
40	91554	P	40	223635	-2	1.19E-10
40	100357	P	40	121266	-2	1.19E-10
40	100357	P	40	227348	-2	1.19E-10
37	100494	P	37	163303	-1	1.21E-10
33	67393	F	33	227556	0	2.78E-10
33	148346	P	33	204264	0	2.78E-10
33	169867	F	33	204264	0	2.78E-10
33	179207	F	33	266215	0	2.78E-10
33	218280	P	33	263408	0	2.78E-10
42	100322	P	42	227381	-3	3.29E-10
39	114136	P	39	251355	-2	4.53E-10
36	44	F	36	9724	-1	4.70E-10
36	44	P	36	42768	-1	4.70E-10
36	121609	F	36	227022	-1	4.70E-10
32	64423	P	32	148422	0	1.11E-09
32	148422	P	32	213242	0	1.11E-09

32	184147	F	32	195578	0	1.11E-09
32	195578	P	32	270129	0	1.11E-09
41	8535	F	41	228525	-3	1.22E-09
41	52397	P	41	228521	-3	1.22E-09
35	6944	P	35	100259	-1	1.83E-09
35	13611	F	35	72413	-1	1.83E-09
35	125003	F	35	160111	-1	1.83E-09
31	25169	F	31	113654	0	4.46E-09
31	72546	F	31	214440	0	4.46E-09
31	82205	P	31	180282	0	4.46E-09
31	84652	P	31	106441	0	4.46E-09
31	84652	P	31	177382	0	4.46E-09
31	101256	P	31	262114	0	4.46E-09
31	134456	F	31	171498	0	4.46E-09
40	75776	P	40	150861	-3	4.53E-09
40	77950	F	40	148284	-3	4.53E-09
40	77950	P	40	204309	-3	4.53E-09
37	13609	F	37	214321	-2	6.52E-09
37	64278	P	37	83275	-2	6.52E-09
37	67379	F	37	160114	-2	6.52E-09
37	98658	F	37	98719	-2	6.52E-09
37	98658	F	37	146048	-2	6.52E-09
37	98658	P	37	167464	-2	6.52E-09
37	98658	F	37	214386	-2	6.52E-09
37	98658	F	37	270230	-2	6.52E-09
37	134398	P	37	148149	-2	6.52E-09
37	157785	P	37	253050	-2	6.52E-09

37	205335	P	37	260509	-2	6.52E-09
34	37434	F	34	37491	-1	7.10E-09
34	77970	P	34	169910	-1	7.10E-09
34	143934	P	34	167414	-1	7.10E-09
39	72681	F	39	232822	-3	1.68E-08
39	91259	P	39	232819	-3	1.68E-08
30	5489	P	30	36139	0	1.78E-08
30	8708	P	30	105660	0	1.78E-08
30	18848	F	30	98658	0	1.78E-08
30	32441	P	30	218082	0	1.78E-08
30	39326	P	30	72546	0	1.78E-08
30	39326	F	30	143806	0	1.78E-08
30	39403	P	30	56642	0	1.78E-08
30	39403	F	30	84652	0	1.78E-08
30	41592	F	30	143939	0	1.78E-08
30	52231	F	30	105660	0	1.78E-08
30	56951	P	30	81353	0	1.78E-08
30	67948	P	30	106928	0	1.78E-08
30	101256	F	30	154355	0	1.78E-08
30	143806	P	30	214440	0	1.78E-08
30	154355	P	30	262115	0	1.78E-08
30	163457	P	30	247055	0	1.78E-08
30	184147	F	30	232819	0	1.78E-08
30	231307	F	30	232494	0	1.78E-08
30	232819	P	30	270131	0	1.78E-08
36	3420	P	36	3420	-2	2.47E-08
36	14560	F	36	226717	-2	2.47E-08

36	180165	F	36	228664	-2	2.47E-08
33	920	F	33	150864	-1	2.76E-08
33	4950	F	33	121087	-1	2.76E-08
33	13613	P	33	39444	-1	2.76E-08
33	36463	F	33	97637	-1	2.76E-08
33	39127	P	33	215580	-1	2.76E-08
33	83027	P	33	121087	-1	2.76E-08
33	97637	F	33	105248	-1	2.76E-08
33	106577	F	33	121087	-1	2.76E-08
33	121087	F	33	177518	-1	2.76E-08
33	180590	P	33	180590	-1	2.76E-08
38	7601	F	38	7643	-3	6.19E-08
38	13502	P	38	97411	-3	6.19E-08
38	27214	P	38	150284	-3	6.19E-08
38	36422	F	38	97595	-3	6.19E-08
38	40387	F	38	247047	-3	6.19E-08
38	57602	P	38	184137	-3	6.19E-08
38	57602	F	38	270133	-3	6.19E-08
38	97595	F	38	105207	-3	6.19E-08
38	98657	F	38	180855	-3	6.19E-08
38	113888	P	38	251598	-3	6.19E-08
35	8624	P	35	42805	-2	9.32E-08
35	42805	F	35	52310	-2	9.32E-08
35	56608	F	35	91263	-2	9.32E-08
35	74983	F	35	258871	-2	9.32E-08
35	179317	F	35	266326	-2	9.32E-08
32	8768	F	32	266763	-1	1.07E-07

32	8831	F	32	68602	-1	1.07E-07
32	28512	P	32	91257	-1	1.07E-07
32	52106	P	32	68602	-1	1.07E-07
32	52169	P	32	266763	-1	1.07E-07
32	72575	F	32	82349	-1	1.07E-07
32	100365	P	32	121266	-1	1.07E-07
32	100365	P	32	227348	-1	1.07E-07
32	180165	F	32	266763	-1	1.07E-07
32	214089	P	32	241371	-1	1.07E-07
37	16996	F	37	174082	-3	2.28E-07
37	57606	P	37	72665	-3	2.28E-07
37	94539	P	37	97398	-3	2.28E-07
37	160114	F	37	227542	-3	2.28E-07
34	7563	F	34	7605	-2	3.52E-07
34	97415	F	34	98727	-2	3.52E-07
34	97415	F	34	146056	-2	3.52E-07
34	97415	P	34	167459	-2	3.52E-07
34	97415	F	34	214394	-2	3.52E-07
34	97415	F	34	270238	-2	3.52E-07
34	228388	P	34	248158	-2	3.52E-07
31	39031	P	31	215671	-1	4.14E-07
31	72546	F	31	180910	-1	4.14E-07
31	151437	P	31	238895	-1	4.14E-07
36	43405	F	36	78003	-3	8.39E-07
36	57594	P	36	232819	-3	8.39E-07
36	72677	F	36	149326	-3	8.39E-07
36	157709	P	36	223543	-3	8.39E-07

36	179845	F	36	266488	-3	8.39E-07
33	8557	P	33	42861	-2	1.32E-06
33	39368	P	33	97416	-2	1.32E-06
33	42861	F	33	52379	-2	1.32E-06
33	64484	F	33	204251	-2	1.32E-06
33	67317	P	33	245845	-2	1.32E-06
33	77950	P	33	264814	-2	1.32E-06
33	77971	F	33	148303	-2	1.32E-06
33	97416	F	33	106474	-2	1.32E-06
33	97416	F	33	177415	-2	1.32E-06
33	97416	P	33	232755	-2	1.32E-06
33	98893	F	33	260814	-2	1.32E-06
33	148422	F	33	249936	-2	1.32E-06
33	197535	F	33	238889	-2	1.32E-06
33	204251	F	33	213303	-2	1.32E-06
33	204251	P	33	249874	-2	1.32E-06
30	5745	F	30	184095	-1	1.60E-06
30	40933	F	30	258877	-1	1.60E-06
30	41441	P	30	184095	-1	1.60E-06
30	55433	F	30	143938	-1	1.60E-06
30	72476	F	30	98658	-1	1.60E-06
30	82343	P	30	98608	-1	1.60E-06
30	84652	P	30	234241	-1	1.60E-06
30	101258	F	30	124849	-1	1.60E-06
30	124846	F	30	154354	-1	1.60E-06
30	132031	F	30	196821	-1	1.60E-06
30	143806	P	30	180910	-1	1.60E-06

30	178782	F	30	265785	-1	1.60E-06
30	180018	F	30	266663	-1	1.60E-06
30	218316	P	30	263375	-1	1.60E-06
30	227487	P	30	245845	-1	1.60E-06
30	268123	P	30	268830	-1	1.60E-06
35	94549	P	35	97390	-3	3.08E-06
35	112580	P	35	124168	-3	3.08E-06
35	115649	F	35	115689	-3	3.08E-06
35	179325	F	35	266334	-3	3.08E-06
35	228291	P	35	248254	-3	3.08E-06
32	41592	P	32	167411	-2	4.97E-06
32	55434	P	32	167411	-2	4.97E-06
32	67379	F	32	125006	-2	4.97E-06
32	67474	F	32	227637	-2	4.97E-06
32	72546	F	32	106519	-2	4.97E-06
32	72546	F	32	177460	-2	4.97E-06
32	72681	F	32	184150	-2	4.97E-06
32	72681	P	32	270126	-2	4.97E-06
32	78555	P	32	79736	-2	4.97E-06
32	82988	F	32	167409	-2	4.97E-06
32	228054	P	32	248485	-2	4.97E-06
32	228664	F	32	266763	-2	4.97E-06
34	66024	P	34	191964	-3	1.12E-05
34	94539	P	34	98652	-3	1.12E-05
31	5744	P	31	270183	-2	1.86E-05
31	39448	F	31	167518	-2	1.86E-05
31	41354	P	31	68030	-2	1.86E-05

31	41564	F	31	180910	-2	1.86E-05
31	41564	F	31	214440	-2	1.86E-05
31	124849	P	31	262112	-2	1.86E-05
31	167384	F	31	191057	-2	1.86E-05
31	167518	P	31	214323	-2	1.86E-05
31	197923	F	31	260793	-2	1.86E-05
31	250296	F	31	250523	-2	1.86E-05
31	250435	F	31	250567	-2	1.86E-05
33	28476	F	33	184120	-3	4.10E-05
33	28476	P	33	270155	-3	4.10E-05
33	43348	F	33	77971	-3	4.10E-05
33	149327	F	33	232819	-3	4.10E-05
33	168105	F	33	212953	-3	4.10E-05
33	169910	F	33	204297	-3	4.10E-05
33	179811	F	33	266454	-3	4.10E-05
33	228029	P	33	248509	-3	4.10E-05
30	4957	F	30	191059	-2	6.98E-05
30	13507	P	30	98665	-2	6.98E-05
30	27472	F	30	145606	-2	6.98E-05
30	39326	P	30	41564	-2	6.98E-05
30	41594	P	30	82990	-2	6.98E-05
30	42777	F	30	52276	-2	6.98E-05
30	55413	F	30	106526	-2	6.98E-05
30	55413	F	30	177467	-2	6.98E-05
30	57600	P	30	195578	-2	6.98E-05
30	64223	P	30	83343	-2	6.98E-05
30	65290	F	30	65314	-2	6.98E-05

30	72678	F	30	195578	-2	6.98E-05
30	75786	P	30	150861	-2	6.98E-05
30	82992	P	30	143939	-2	6.98E-05
30	83023	P	30	191059	-2	6.98E-05
30	86805	P	30	238895	-2	6.98E-05
30	91268	P	30	195578	-2	6.98E-05
30	97421	F	30	180870	-2	6.98E-05
30	98666	F	30	98727	-2	6.98E-05
30	98666	F	30	146056	-2	6.98E-05
30	98666	P	30	167463	-2	6.98E-05
30	98666	F	30	214394	-2	6.98E-05
30	98666	F	30	270238	-2	6.98E-05
30	105660	P	30	228604	-2	6.98E-05
30	106519	P	30	143806	-2	6.98E-05
30	106584	F	30	191059	-2	6.98E-05
30	140320	F	30	215176	-2	6.98E-05
30	143806	P	30	177460	-2	6.98E-05
30	168105	P	30	214296	-2	6.98E-05
30	177525	F	30	191059	-2	6.98E-05
32	46782	F	32	221509	-3	1.49E-04
32	49679	P	32	60178	-3	1.49E-04
32	72681	P	32	91263	-3	1.49E-04
32	98831	P	32	121668	-3	1.49E-04
32	106120	F	32	259914	-3	1.49E-04
32	114062	P	32	251438	-3	1.49E-04
32	125006	F	32	227542	-3	1.49E-04
32	178636	F	32	265641	-3	1.49E-04

32	228168	P	32	248377	-3	1.49E-04
32	228318	P	32	248230	-3	1.49E-04
31	922	P	31	75780	-3	5.41E-04
31	25166	P	31	167416	-3	5.41E-04
31	30488	F	31	184297	-3	5.41E-04
31	43416	P	31	64494	-3	5.41E-04
31	43416	P	31	213313	-3	5.41E-04
31	43416	F	31	249866	-3	5.41E-04
31	47190	F	31	221920	-3	5.41E-04
31	55439	P	31	82986	-3	5.41E-04
31	56457	F	31	187511	-3	5.41E-04
31	57603	P	31	83213	-3	5.41E-04
31	72413	P	31	167518	-3	5.41E-04
31	80040	P	31	161680	-3	5.41E-04
31	91650	F	31	223914	-3	5.41E-04
31	113599	F	31	184080	-3	5.41E-04
31	143869	P	31	180860	-3	5.41E-04
30	8549	F	30	228539	-3	1.95E-03
30	13373	F	30	187514	-3	1.95E-03
30	13691	F	30	179609	-3	1.95E-03
30	18848	F	30	180856	-3	1.95E-03
30	20651	P	30	205076	-3	1.95E-03
30	23385	F	30	81844	-3	1.95E-03
30	26332	P	30	134459	-3	1.95E-03
30	28512	F	30	149336	-3	1.95E-03
30	28512	F	30	232828	-3	1.95E-03
30	28516	P	30	56600	-3	1.95E-03

30	29304	P	30	231491	-3	1.95E-03
30	36361	F	30	105134	-3	1.95E-03
30	36361	P	30	269571	-3	1.95E-03
30	36422	P	30	170581	-3	1.95E-03
30	39373	P	30	98665	-3	1.95E-03
30	39448	P	30	146001	-3	1.95E-03
30	56613	P	30	184147	-3	1.95E-03
30	56613	F	30	270131	-3	1.95E-03
30	56804	P	30	210687	-3	1.95E-03
30	57606	F	30	91274	-3	1.95E-03
30	57606	P	30	149321	-3	1.95E-03
30	66356	P	30	76578	-3	1.95E-03
30	67320	F	30	227487	-3	1.95E-03
30	72407	F	30	234310	-3	1.95E-03
30	72415	F	30	146002	-3	1.95E-03
30	83217	F	30	184147	-3	1.95E-03
30	83217	P	30	270131	-3	1.95E-03
30	84562	P	30	180907	-3	1.95E-03
30	91281	P	30	184134	-3	1.95E-03
30	91281	F	30	270144	-3	1.95E-03
30	96518	P	30	99955	-3	1.95E-03
30	97414	F	30	98665	-3	1.95E-03
30	98667	F	30	106474	-3	1.95E-03
30	98667	F	30	177415	-3	1.95E-03
30	98667	P	30	232758	-3	1.95E-03
30	105207	P	30	170581	-3	1.95E-03
30	106409	F	30	180860	-3	1.95E-03

30	112540	P	30	124213	-3	1.95E-03
30	134361	F	30	217295	-3	1.95E-03
30	134426	F	30	171461	-3	1.95E-03
30	137225	F	30	251089	-3	1.95E-03
30	141211	F	30	141687	-3	1.95E-03
30	146002	F	30	214325	-3	1.95E-03
30	149327	F	30	184147	-3	1.95E-03
30	149327	F	30	195578	-3	1.95E-03
30	149327	P	30	270131	-3	1.95E-03
30	157534	F	30	157592	-3	1.95E-03
30	170582	F	30	269499	-3	1.95E-03
30	178296	F	30	265342	-3	1.95E-03
30	179823	F	30	266466	-3	1.95E-03
30	220218	F	30	220355	-3	1.95E-03
30	228069	P	30	248472	-3	1.95E-03
30	228177	P	30	248370	-3	1.95E-03
30	234314	P	30	262147	-3	1.95E-03

Table S4 The DNA transfer in the mitochondrial genome of *I. batatas*

Number	identities	length	start	end	start	end	evalue	mitogene
1	80.863	371	68116	68477	55849	56202	4.98E-71	trnW-CCA
2	79.688	192	46648	46837	56763	56945	8.82E-29	IGS(trnW-CCA-ccmFc)
3	100	28	8133	8160	56832	56805	5.46E-06	IGS(trnW-CCA-ccmFc)
4	96.61	59	31944	32002	68740	68682	6.92E-20	IGS(rps12-nad7)
5	91.447	152	35917	36063	101952	102103	1.43E-51	IGS(trnK-UUU-matR)
6	96.375	331	399	729	104760	105089	7.4E-154	IGS(trnK-UUU-matR)
7	89.764	127	31416	31542	111041	110916	8.7E-39	trnD-GUC
8	99.769	1299	140340	141638	111586	112884	0	IGS(trnD-GUC-trnE-UUC)
9	96.88	3462	42340	45763	117129	113687	0	IGS(trnD-GUC-trnE-UUC)
10	80.992	121	39831	39951	117452	117332	2.49E-19	IGS(trnD-GUC-trnE-UUC)

11	80.992	121	42055	42175	119676	119556	2.49E-19	IGS(trnD-GUC-trnE-UUC)
12	99.216	2935	39398	42332	120109	117194	0	IGS(trnD-GUC-trnE-UUC)
13	88.852	305	36884	37187	120379	120098	1.34E-96	IGS(trnD-GUC-trnE-UUC)
14	91.31	1473	81313	82756	154382	155832	0	IGS(rpl16-mttB)
15	92.839	1173	82756	83906	155780	156941	0	IGS(rpl16-mttB)
16	99.636	549	100246	100794	168105	167557	0	IGS(mttB-nad4L)
17	74.074	891	146560	147423	179402	178543	2.27E-84	rrn18
18	93.59	78	53994	54071	197227	197150	1.91E-25	trnM-CAU
19	96.296	54	84943	84996	240437	240384	4.16E-17	IGS(rps4-atp6)
20	84.534	944	90885	91812	241282	242209	0	IGS(rps4-atp6)
21	82.799	593	92235	92818	242742	243315	3.5E-142	IGS(rps4-atp6)
22	90.402	1021	154854	155860	244810	243802	0	IGS(rps4-atp6)
23	96.61	118	59840	59957	24572	24584	2.38E	atp6

					9	6	-49	
24	94.471	416	55658	56067	245731	245316	0	IGS(rps4-atp6)
25	96.296	81	109501	109581	261401	261481	1.9E-30	trnN-GUU
26	99.245	2912	145006	147902	267979	265069	0	IGS(trnN-GUU-nad5)

Table S5 The RNA editing events prediction in the *I. batatas*.

gene	Position	Coverage	Reference	Editing efficiency	Edited number	Alternative
atp1	1039	414	C	0.9806	406	U
atp1	1244	448	C	0.029	13	U
atp1	1247	462	C	0.0735	34	U
atp1	1253	464	A	0.028	13	G
atp1	1414	727	C	0.2819	205	U
atp1	1489	708	C	0.0169	12	U
atp1	1490	675	C	0.9614	649	U
atp4	50	53	C	0.9622	51	U
atp4	62	49	C	0.9183	45	U
atp4	109	59	C	0.8983	53	U
atp4	206	106	C	0.9433	100	U
atp4	218	105	C	0.9428	99	U
atp4	219	106	C	0.1132	12	U
atp4	239	114	C	0.9736	111	U
atp4	242	114	C	0.9824	112	U
atp4	297	125	C	0.144	18	U
atp4	386	145	C	0.9931	144	U
atp4	398	145	C	0.9931	144	U
atp6	27	40	C	0.375	15	A
atp6	29	36	C	0.3055	11	A
atp6	454	31	C	1	31	U
atp6	533	45	C	0.9111	41	U
atp6	584	50	C	0.88	44	U
atp6	590	50	C	0.9	44	U
atp6	641	52	C	0.8653	45	U
atp6	653	54	C	0.8888	48	U
atp6	671	54	C	0.9074	49	U
atp6	679	54	C	0.9444	51	U
atp6	686	50	C	0.96	48	U
atp6	687	50	C	0.64	32	U
atp6	877	30	C	0.8666	26	U
atp6	880	30	C	1	30	U
atp6	902	34	C	0.9117	31	U
atp6	944	26	C	1	26	U
atp6	1073	21	C	0.9523	20	U
atp6	1081	21	C	1	21	U
atp6	1088	22	C	0.9545	21	U
atp6	1095	22	C	0.6363	14	U
atp6	1097	22	C	0.9545	21	U

atp6	1135	25	C	0.88	22	U
atp8	39	294	C	0.0544	16	U
atp8	47	296	C	0.7736	229	U
atp8	58	318	C	0.5377	171	U
atp8	76	319	C	0.5454	174	U
atp8	165	458	C	0.0458	21	U
atp8	219	403	C	0.4143	167	U
atp8	449	222	C	0.7657	170	U
atp9	20	2834	C	0.9844	2789	U
atp9	50	2626	C	0.9904	2601	U
atp9	57	2570	A	0.0077	10	G
atp9	60	2540	T	0.007	15	A
atp9	64	2589	G	0.0065	16	A
atp9	70	2569	A	0.0062	16	C
atp9	78	2740	C	0.0062	17	U
atp9	81	2605	C	0.9696	2525	U
atp9	82	2608	C	0.9846	2568	U
atp9	90	2756	C	0.169	466	U
atp9	92	2798	C	0.9853	2757	U
atp9	97	2844	C	0.0295	84	U
atp9	104	2641	T	0.0098	22	G
atp9	106	2261	G	0.0044	10	A
atp9	182	2114	C	0.9952	2104	U
atp9	190	2051	C	0.0258	53	U
atp9	191	1956	C	0.9867	1924	U
atp9	202	1758	T	0.0113	10	C
atp9	212	1714	C	0.9889	1695	U
atp9	216	1705	C	0.0381	65	U
atp9	223	1697	C	0.9811	1664	U
atp9	227	1679	C	0.0119	20	U
atp9	241	1344	A	0.0252	33	G
atp9	251	838	T	0.0381	27	G
ccmB	304	18	C	0.7777	14	U
ccmB	424	15	C	0.6666	10	U
ccmB	428	15	C	0.6666	10	U
ccmB	467	25	C	0.56	14	U
ccmB	485	28	C	0.6428	18	U
ccmB	494	28	C	0.5357	15	U
ccmB	503	28	C	0.5714	16	U
ccmB	512	27	C	0.5555	15	U
ccmB	514	26	C	0.5769	15	U
ccmB	551	31	C	0.5483	17	U

ccmB	554	30	C	0.5333	16	U
ccmB	566	31	C	0.5483	17	U
ccmB	569	29	C	0.5172	15	U
ccmB	572	30	C	0.5333	16	U
ccmB	576	30	C	0.3666	11	U
ccmC	76	27	C	0.9259	25	U
ccmC	84	31	C	0.3225	10	U
ccmC	103	31	C	0.8387	26	U
ccmC	115	32	C	0.875	28	U
ccmC	133	31	C	0.9677	30	U
ccmC	179	36	C	0.8888	32	U
ccmC	184	31	C	0.9354	29	U
ccmC	299	20	C	0.95	19	U
ccmC	399	94	C	0.7021	66	U
ccmC	400	94	C	0.6808	64	U
ccmC	418	80	C	0.1374	11	U
ccmC	421	104	C	1	104	U
ccmC	436	115	C	0.9913	114	U
ccmC	446	120	C	0.9833	118	U
ccmC	451	123	C	0.9674	119	U
ccmC	458	131	C	0.9465	124	U
ccmC	463	136	C	0.9411	128	U
ccmC	467	135	C	0.9333	126	U
ccmC	473	143	C	0.944	134	U
ccmC	477	148	C	0.1689	25	U
ccmC	497	151	C	0.9536	144	U
ccmC	548	131	C	0.9389	123	U
ccmC	568	143	C	0.93	133	U
ccmC	575	139	C	0.9208	128	U
ccmC	605	97	C	0.9484	92	U
ccmC	606	98	C	0.6326	62	U
ccmC	607	99	C	0.1111	11	U
ccmC	608	97	C	0.969	94	U
ccmC	614	100	C	0.95	95	U
ccmC	618	99	C	0.5656	56	U
ccmC	619	100	C	0.96	96	U
ccmC	624	98	C	0.9387	92	U
ccmC	650	75	C	0.9333	70	U
ccmC	655	72	C	0.1805	13	U
ccmC	656	72	C	0.9166	66	U
ccmC	673	60	C	0.85	51	U
ccmFc	29	122	C	0.2786	31	U

ccmFc	62	157	C	0.2292	36	A
ccmFc	72	157	C	0.4076	64	U
ccmFc	74	156	C	0.2948	46	U
ccmFc	125	129	C	0.5658	73	U
ccmFc	144	118	C	0.5932	70	U
ccmFc	168	115	C	0.7217	83	U
ccmFc	173	101	C	0.7722	78	U
ccmFc	177	103	C	0.4563	47	U
ccmFc	413	65	C	0.8	52	U
ccmFc	448	281	C	0.0355	10	U
ccmFc	905	31	C	0.5806	18	U
ccmFc	935	32	C	0.5937	19	U
ccmFn	38	35	C	0.5142	18	U
ccmFn	98	27	C	0.5925	16	U
ccmFn	137	23	C	0.7391	17	U
ccmFn	142	23	C	0.7826	18	U
ccmFn	151	25	C	0.64	16	U
ccmFn	248	16	C	0.6875	11	U
ccmFn	256	14	C	0.7142	10	U
ccmFn	365	14	C	0.8571	12	U
ccmFn	372	14	C	0.7857	11	U
ccmFn	767	20	C	0.95	19	U
ccmFn	779	21	C	0.9523	20	U
ccmFn	794	22	C	0.8181	18	U
ccmFn	943	16	C	0.8125	13	U
ccmFn	1261	11	C	0.909	10	U
ccmFn	1289	11	C	0.909	10	U
ccmFn	1321	12	C	0.8333	10	U
ccmFn	1390	12	C	0.8333	10	U
ccmFn	1433	12	C	0.8333	10	U
ccmFn	1457	15	C	0.8	12	U
ccmFn	1469	15	C	0.8	12	U
ccmFn	1504	13	C	0.7692	10	U
cob	298	61	C	0.9836	60	U
cob	325	68	C	0.9264	63	U
cob	568	44	C	0.8636	38	U
cob	680	33	C	0.9696	32	U
cob	808	43	C	0.9302	40	U
cob	908	37	C	0.8378	31	U
cob	982	58	C	0.862	50	U
cob	1084	104	C	0.9134	95	U
cob	1160	78	C	0.2948	23	U

cox1	11	136	C	0.8676	118	U
cox1	15	137	C	0.2773	38	U
cox1	242	89	C	0.9325	83	U
cox1	254	83	C	0.8795	73	U
cox1	265	78	C	0.2564	20	U
cox1	352	68	C	0.8235	56	U
cox1	443	102	C	0.8725	89	U
cox1	452	98	C	0.9387	92	U
cox1	551	137	C	0.9854	135	U
cox1	590	126	C	0.9523	120	U
cox1	668	81	C	0.9012	72	U
cox1	715	95	C	0.9684	92	U
cox1	1186	90	C	0.9444	85	U
cox1	1405	101	C	0.9108	92	U
cox1	1433	91	C	0.912	83	U
cox1	1489	91	C	0.8791	80	U
cox1	1499	81	C	0.8765	71	U
cox2	37	118	C	0.9152	108	U
cox2	38	116	C	0.862	100	U
cox2	71	98	C	1	98	U
cox2	138	86	C	0.1395	12	U
cox2	161	91	C	0.978	89	U
cox2	253	103	C	0.9708	100	U
cox2	278	123	C	0.9268	114	U
cox2	379	115	C	0.9478	109	U
cox2	383	111	G	0.9459	105	C
cox2	443	150	C	0.98	147	U
cox2	461	172	C	0.9709	167	U
cox2	476	170	C	0.947	161	U
cox2	557	235	C	0.9872	231	U
cox2	574	223	C	0.0807	18	U
cox2	632	199	C	0.9698	193	U
cox2	666	174	C	0.2011	35	U
cox2	742	138	C	0.9202	127	U
cox3	245	283	C	0.9328	264	U
cox3	298	321	C	0.894	286	U
cox3	304	314	C	0.8375	260	U
cox3	311	232	C	0.875	203	U
cox3	314	236	C	0.8432	199	U
cox3	419	208	C	0.9615	200	U
cox3	422	211	C	0.872	184	U
cox3	512	233	C	0.8841	206	U

cox3	653	153	C	0.9738	149	U
cox3	702	196	T	0.153	30	C
cox3	749	1859	T	0.9338	1733	G
cox3	754	1616	C	0.9622	1555	U
cox3	762	1626	C	0.0067	11	U
cox3	764	1616	C	0.6875	1111	U
cox3	776	155	T	0.4774	74	G
matR	193	62	C	0.8709	54	U
matR	235	58	C	0.8965	52	U
matR	236	58	C	0.3275	19	U
matR	326	50	C	0.6799	34	U
matR	413	48	C	0.9375	45	U
matR	1545	47	C	0.4255	20	U
matR	1679	63	C	0.8095	51	U
matR	1700	68	C	0.8235	56	U
matR	1720	69	C	0.8985	62	U
matR	1734	73	C	0.8904	65	U
matR	1787	63	C	0.9206	58	U
matR	1844	44	C	0.8636	38	U
mttB	431	12	C	0.8333	10	U
nad1	215	27	C	0.9629	26	U
nad1	265	26	C	0.8461	22	U
nad1	307	24	C	0.8333	20	U
nad1	308	24	C	0.8333	20	U
nad1	436	51	C	0.5294	27	U
nad1	492	32	C	0.5937	19	U
nad1	493	36	C	0.8333	30	U
nad1	500	39	C	0.641	25	U
nad1	536	39	C	0.7179	28	U
nad1	580	47	C	0.8723	41	U
nad1	635	52	C	0.9615	50	U
nad1	674	28	C	0.9285	26	U
nad1	734	15	C	0.6666	10	U
nad1	740	15	C	0.7333	11	U
nad1	755	16	C	0.9375	15	U
nad1	779	19	C	0.7368	14	U
nad1	898	16	C	1	16	U
nad1	928	19	C	0.9473	18	U
nad1	937	20	C	0.95	19	U
nad2	223	13	C	0.8461	11	U
nad2	252	17	C	0.5882	10	U
nad2	308	24	C	0.875	21	U

nad2	341	22	C	0.8636	19	U
nad2	356	23	C	0.6956	16	U
nad2	361	23	C	0.7391	17	U
nad2	367	22	C	0.9545	21	U
nad2	401	20	C	0.95	19	U
nad2	428	16	C	0.9375	15	U
nad2	497	18	C	0.8333	15	U
nad2	523	13	C	0.7692	10	U
nad2	668	18	C	0.6666	12	U
nad2	788	22	C	0.6363	14	U
nad2	800	22	C	0.5909	13	U
nad2	809	23	C	0.5217	12	U
nad2	928	15	C	0.6666	10	U
nad2	958	13	C	0.8461	11	U
nad2	962	15	C	0.7333	11	U
nad2	1028	24	C	0.5	12	U
nad2	1246	31	C	0.8709	27	U
nad2	1247	30	C	0.8666	26	U
nad2	1276	38	C	0.8684	33	U
nad2	1298	48	C	0.8541	41	U
nad2	1400	69	C	0.971	67	U
nad2	1404	70	C	0.1571	11	U
nad2	1408	70	C	0.9857	69	U
nad2	1409	70	C	0.9714	68	U
nad2	1457	51	C	1	51	U
nad3	5	36	C	0.4166	15	U
nad3	39	150	C	0.1266	19	U
nad3	44	145	C	0.8137	118	U
nad3	61	150	C	0.6733	101	U
nad3	62	148	C	0.7364	109	U
nad3	80	161	C	0.8322	134	U
nad3	146	196	C	0.8979	176	U
nad3	208	236	C	0.805	190	U
nad3	209	234	C	0.8205	192	U
nad3	215	246	C	0.8089	199	U
nad3	230	250	C	0.856	214	U
nad3	231	250	C	0.272	68	U
nad3	266	220	C	0.8181	180	U
nad3	275	230	C	0.8565	197	U
nad3	317	189	C	0.8359	158	U
nad3	344	166	C	0.771	128	U
nad3	349	163	C	0.8098	132	U

nad4	29	82	C	0.9268	76	U
nad4	74	93	C	0.8387	78	U
nad4	77	103	C	0.7669	79	U
nad4	107	106	C	0.8773	92	U
nad4	124	118	C	0.7627	90	U
nad4	154	118	C	0.805	95	U
nad4	158	119	C	0.8655	103	U
nad4	197	105	C	0.8857	93	U
nad4	362	88	C	0.4318	38	U
nad4	368	73	C	0.9041	66	U
nad4	376	74	C	0.9189	68	U
nad4	433	69	C	0.6956	48	U
nad4	436	69	C	0.9565	66	U
nad4	437	68	C	0.8382	57	U
nad4	608	125	C	0.952	119	U
nad4	659	121	C	0.6859	83	U
nad4	767	145	C	0.7448	108	U
nad4	836	160	C	0.6625	106	U
nad4	887	139	C	0.6187	86	U
nad4	977	125	C	0.256	26	U
nad4	1006	128	C	0.7421	95	U
nad4	1009	130	C	0.2307	30	U
nad4	1010	131	C	0.7786	102	U
nad4	1016	135	C	0.6814	92	U
nad4	1109	196	C	0.8265	162	U
nad4	1129	184	C	0.7989	147	U
nad4	1132	189	C	0.7777	147	U
nad4	1148	187	C	0.7326	137	U
nad4	1151	186	C	0.7634	142	U
nad4	1152	185	C	0.1135	21	U
nad4	1172	190	C	0.8368	159	U
nad4	1194	188	C	0.0531	10	U
nad4	1307	223	C	0.843	188	U
nad4	1355	178	C	0.9775	174	U
nad4	1373	164	C	0.8536	140	U
nad4	1406	170	C	0.9117	155	U
nad4	1418	178	C	0.8483	151	U
nad4	1434	174	C	0.8793	153	U
nad4	1439	164	C	0.8475	139	U
nad4L	86	13	C	0.8461	11	U
nad4L	95	13	C	0.923	12	U
nad4L	100	14	C	0.8571	12	U

nad4L	110	16	C	0.9375	15	U
nad4L	131	16	C	0.9375	15	U
nad4L	158	18	C	1	18	U
nad4L	179	22	C	1	22	U
nad4L	188	22	C	1	22	U
nad4L	197	22	C	1	22	U
nad4L	281	24	C	0.9583	23	U
nad5	155	31	C	0.4838	15	U
nad5	227	24	C	0.5	12	U
nad5	229	23	G	0.5217	12	U
nad5	242	25	C	0.92	23	U
nad5	358	30	C	0.9333	28	U
nad5	359	31	C	0.9677	30	U
nad5	374	29	C	0.7241	21	U
nad5	398	28	C	0.7857	22	U
nad5	506	33	C	0.6363	21	U
nad5	539	35	C	0.8857	31	U
nad5	548	36	C	0.7222	26	U
nad5	598	32	C	0.7812	25	U
nad5	608	37	C	0.5405	20	U
nad5	629	53	C	0.6037	32	U
nad5	713	30	C	0.9	27	U
nad5	725	28	C	0.8928	25	U
nad5	835	25	C	1	25	U
nad5	1310	31	C	0.4193	13	U
nad5	1469	27	C	0.9629	26	U
nad5	1529	33	C	0.8484	28	U
nad5	1547	25	C	0.8	20	U
nad5	1559	27	C	0.8888	24	U
nad5	1568	33	C	0.7272	24	U
nad5	1589	37	C	0.5945	22	U
nad5	1874	28	C	0.9285	26	U
nad5	1895	33	C	0.9393	31	U
nad5	1897	32	C	0.9375	30	U
nad5	1937	34	C	0.9117	31	U
nad6	26	51	C	0.745	38	U
nad6	88	65	C	0.8769	57	U
nad6	89	65	C	0.8615	56	U
nad6	95	70	C	0.8142	57	U
nad6	103	67	C	0.8358	56	U
nad6	161	68	C	0.8676	59	U
nad6	569	325	C	0.9969	324	U

nad7	38	52	C	0.5	26	U
nad7	45	54	C	0.3518	19	U
nad7	77	66	C	0.7272	48	U
nad7	83	65	C	0.5538	36	U
nad7	137	45	C	0.6888	31	U
nad7	200	28	C	0.8928	25	U
nad7	244	44	C	0.75	33	U
nad7	251	45	C	0.6	27	U
nad7	315	48	C	0.2708	13	U
nad7	316	49	C	0.8163	40	U
nad7	335	53	C	0.7547	40	U
nad7	344	54	C	0.5555	30	U
nad7	383	48	C	0.8333	40	U
nad7	578	51	C	0.8235	42	U
nad7	724	29	C	0.8275	24	U
nad7	739	22	C	0.5	11	U
nad7	740	23	C	0.5217	12	U
nad7	769	18	C	0.6666	12	U
nad7	926	24	C	0.5416	13	U
nad7	973	23	C	0.6956	16	U
nad7	1050	30	C	0.5333	16	U
nad7	1057	32	C	0.75	24	U
nad7	1079	32	C	0.75	24	U
nad7	1088	32	C	0.7187	23	U
nad7	1103	41	C	0.7804	32	U
nad7	1124	40	C	0.8	32	U
nad7	1166	29	C	0.8965	26	U
nad9	92	166	C	0.2469	41	U
nad9	112	85	C	0.4	34	U
nad9	167	125	C	0.272	34	U
nad9	270	141	C	0.0851	12	U
nad9	298	94	C	0.3191	30	U
nad9	328	93	C	0.4623	43	U
nad9	368	75	C	0.24	18	U
nad9	398	68	C	0.7794	53	U
nad9	439	57	C	0.3333	19	U
rpl10	83	70	C	0.9	63	U
rpl10	101	71	C	0.7183	51	U
rpl10	133	219	C	0.1369	30	U
rpl10	134	219	C	0.1826	40	U
rpl10	180	74	C	0.1351	10	U
rpl10	239	55	C	0.5818	32	U

rpl16	6	1136	C	1	1134	U
rpl16	19	1874	C	0.017	32	U
rpl16	35	2559	C	0.0293	75	U
rpl16	37	2580	C	0.9922	2558	U
rpl16	58	3392	C	0.005	17	U
rpl16	92	4138	C	0.0043	17	U
rpl16	97	4111	C	0.015	60	U
rpl16	101	4203	C	0.0045	14	U
rpl16	118	4592	C	0.0559	256	U
rpl16	149	5047	C	0.0065	32	U
rpl16	150	5076	G	0.0023	11	A
rpl16	183	5968	C	0.0537	311	U
rpl16	184	5942	A	0.0025	13	G
rpl16	185	5782	C	0.998	5758	U
rpl16	201	5831	T	0.0075	32	C
rpl16	204	5812	T	0.0029	14	C
rpl16	217	6031	C	0.0018	10	U
rpl16	238	5686	C	0.0031	12	U
rpl16	249	5716	C	0.3469	1981	U
rpl16	258	5582	C	0.0073	41	U
rpl16	262	5559	C	0.007	36	U
rpl16	272	5474	G	0.0025	14	A
rpl16	273	5480	C	0.0031	16	U
rpl16	276	5408	A	0.0042	12	C
rpl16	282	5499	C	0.0025	10	U
rpl16	294	5469	T	0.004	17	C
rpl16	315	5972	C	0.0051	30	U
rpl16	321	5904	T	0.0016	10	C
rpl16	324	5933	C	0.0716	425	U
rpl16	325	5947	C	0.0052	28	U
rpl16	326	5970	C	0.006	32	U
rpl16	332	6127	C	0.0173	106	U
rpl16	356	6091	T	0.0021	10	C
rpl16	360	6231	A	0.0166	76	G
rpl16	361	6294	A	0.0041	20	G
rpl16	373	6413	A	0.0023	10	G
rpl16	376	6409	C	0.002	12	U
rpl16	377	6386	C	0.0081	51	U
rpl16	402	6409	C	0.7178	4600	U
rpl16	414	6457	C	0.0027	15	U
rpl16	429	6213	T	0.0188	53	C
rpl16	432	5789	T	0.0057	20	C

rpl16	450	5924	T	0.004	22	C
rpl16	451	5975	C	0.0025	14	U
rpl16	488	6138	C	0.9876	6062	U
rpl16	491	6093	C	0.0114	68	U
rpl16	495	6200	C	0.0432	265	U
rpl16	505	6160	C	0.0873	537	U
rpl16	511	5977	T	0.0026	12	C
rpl5	35	41	C	0.8536	35	U
rpl5	47	39	C	0.923	36	U
rpl5	59	43	C	0.8604	37	U
rpl5	64	42	C	0.8095	34	U
rpl5	92	48	C	0.8958	43	U
rpl5	161	38	C	0.9473	36	U
rpl5	441	51	C	0.2549	13	U
rpl5	509	34	C	0.8823	30	U
rpl5	512	35	C	0.9714	34	U
rps10	16	80	C	0.8625	68	U
rps10	238	162	C	0.8703	141	U
rps10	302	122	C	0.9016	110	U
rps10	331	120	C	0.9	108	U
rps12	25	270	C	0.2222	60	U
rps12	84	364	C	0.0686	25	U
rps12	102	360	C	0.1083	39	U
rps12	103	361	C	0.2077	75	U
rps12	104	359	C	0.9192	330	U
rps12	146	367	C	0.9727	357	U
rps12	196	349	C	0.957	334	U
rps12	269	282	C	0.8439	238	U
rps12	284	247	C	0.8623	213	U
rps13	26	101	C	0.3366	34	U
rps13	31	94	T	0.5744	54	G
rps13	32	93	C	0.5698	53	U
rps13	33	92	C	0.576	53	A
rps13	34	91	G	0.5824	53	A
rps13	38	94	A	0.5744	54	C
rps13	50	106	T	0.6132	65	G
rps13	56	111	C	0.1441	16	U
rps13	57	112	A	0.625	70	U
rps13	78	126	A	0.619	78	C
rps13	83	125	A	0.592	74	U
rps13	95	124	A	0.6048	75	U
rps13	100	126	C	0.3571	45	U

rps13	287	54	C	0.7037	37	U
rps19	84	1252	C	0.0119	14	U
rps19	100	1267	C	0.0078	10	U
rps19	116	1343	C	0.0245	32	U
rps19	119	1349	C	0.0081	10	U
rps19	126	1363	C	0.0124	17	U
rps19	138	1367	C	0.8317	1137	U
rps19	163	1335	C	0.8838	1180	U
rps19	164	1339	C	0.8894	1191	U
rps19	183	1369	T	0.0138	19	C
rps19	199	1723	C	0.0882	152	U
rps19	240	1678	T	0.2902	487	C
rps19	258	1577	A	0.305	481	G
rps19	263	1357	T	0.3507	468	G
rps19	264	1343	A	0.0089	10	G
rps19	271	1321	G	0.3489	456	A
rps1	23	27	C	0.8148	22	U
rps1	158	14	C	0.7142	10	U
rps3	7	952	C	0.0661	62	U
rps3	92	475	C	0.9768	463	U
rps3	100	452	C	0.0221	10	U
rps3	126	534	C	0.0973	52	U
rps3	131	525	C	0.019	10	U
rps3	227	636	C	0.0172	11	U
rps3	320	573	C	0.1134	63	U
rps3	324	588	C	0.2261	133	U
rps3	518	851	C	0.913	776	U
rps3	719	802	C	0.9638	773	U
rps3	762	716	C	0.0223	16	U
rps3	943	877	C	0.0672	59	U
rps3	1037	822	C	0.7992	657	U
rps3	1261	1849	C	0.007	13	U
rps3	1262	1877	C	0.0724	136	U
rps3	1307	2318	C	0.0133	30	U
rps3	1338	2499	C	0.0116	29	U
rps3	1349	2531	C	0.0086	22	U
rps3	1372	2728	C	0.0278	76	U
rps3	1382	2720	C	0.0176	46	U
rps3	1384	2720	C	0.0577	157	U
rps3	1385	2725	C	0.9944	2710	U
rps3	1412	2845	C	0.9936	2825	U
rps3	1430	2732	C	0.012	31	U

rps3	1435	2809	C	0.0188	52	U
rps3	1440	2735	C	0.0164	45	U
rps3	1464	2586	T	0.0077	18	C
rps3	1468	2619	C	0.0064	16	U
rps3	1482	2635	A	0.0053	12	G
rps3	1484	2651	A	0.0067	14	G
rps3	1490	2465	T	0.0304	62	G
rps3	1492	2713	G	0.0121	33	U
rps3	1493	2928	A	0.0058	10	G
rps3	1499	3448	T	0.0043	10	G
rps3	1501	3535	C	0.0067	20	U
rps3	1506	3762	A	0.0098	15	C
rps3	1514	3905	C	0.0048	19	U
rps3	1519	3938	C	0.0043	17	U
rps3	1532	4069	C	0.0036	13	U
rps3	1547	4239	C	0.0061	26	U
rps3	1554	4224	C	0.0033	13	U
rps3	1557	4266	A	0.0089	23	G
rps3	1574	4215	C	0.0251	105	U
rps3	1576	4207	C	0.9933	4179	U
rps3	1609	3953	C	0.9984	3946	U
rps3	1613	3947	C	0.003	11	U
rps3	1622	3935	C	0.0218	86	U
rps3	1638	3527	C	0.0345	122	U
rps3	1640	3464	C	0.9919	3435	U
rps3	1646	2699	T	0.0118	32	C
rps3	1648	2646	A	0.0105	13	G
rps3	1656	2071	G	0.0053	11	A
rps3	1657	1700	A	0.0217	21	G
rps3	1658	1460	T	0.0232	19	G
rps4	38	26	C	0.5	13	U
rps4	266	46	C	0.7173	33	U
rps4	278	43	C	0.4651	20	U
rps4	290	40	C	0.85	34	U
rps4	335	48	C	0.9375	43	U
rps4	399	41	C	0.2682	11	U
rps4	468	52	C	0.1923	10	U
rps4	671	49	C	0.4081	20	U
rps4	800	38	C	0.3421	13	U
rps4	811	39	C	0.7948	31	U
rps4	821	38	C	0.3157	12	U
rps4	836	37	C	0.5405	20	U

rps4	887	52	C	0.4807	25	U
rps4	901	53	C	0.6603	35	U
sdh4	29	65	C	0.9846	64	U
sdh4	39	82	C	0.9024	74	U
sdh4	132	205	C	0.0975	20	U
sdh4	153	226	C	0.761	172	U
sdh4	154	225	C	0.7511	169	U
sdh4	200	258	C	0.7054	182	U
sdh4	209	266	C	0.6165	164	U
sdh4	265	258	C	0.9069	234	U
sdh4	354	134	C	0.4701	63	U
sdh4	359	128	C	0.9609	123	U

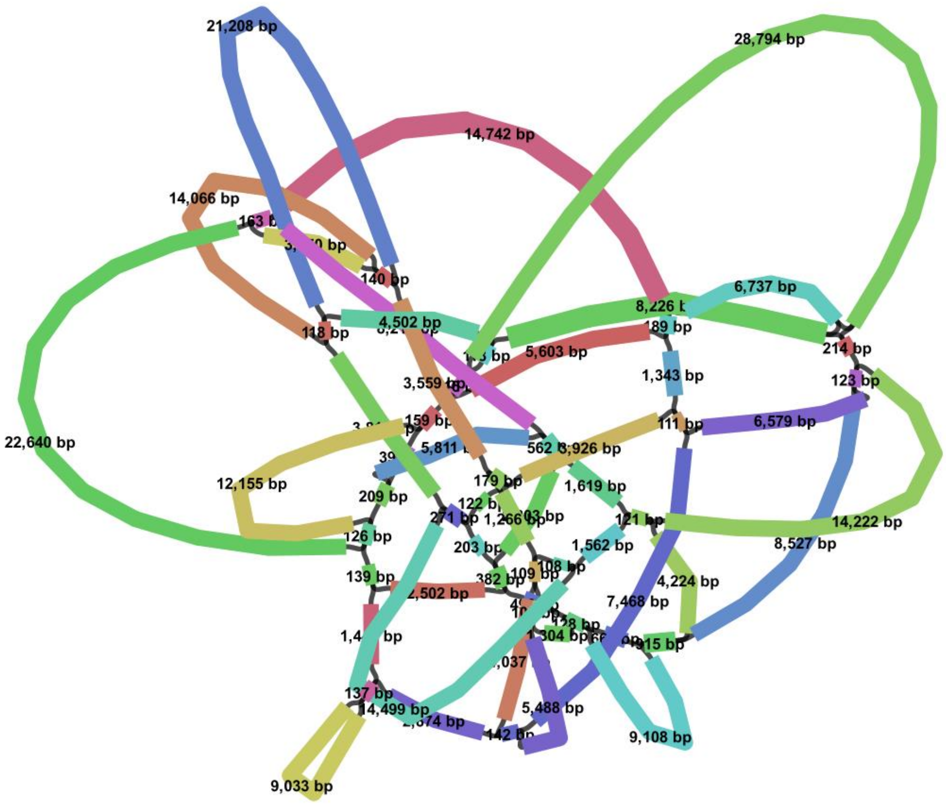

Figure S1. The graph-based mitochondrial genome of *I. batatas.* The graph results were visualized with Bange. The colorful line segments represent different mitochondrial genome segments and the black lines represent potential linkage patterns.

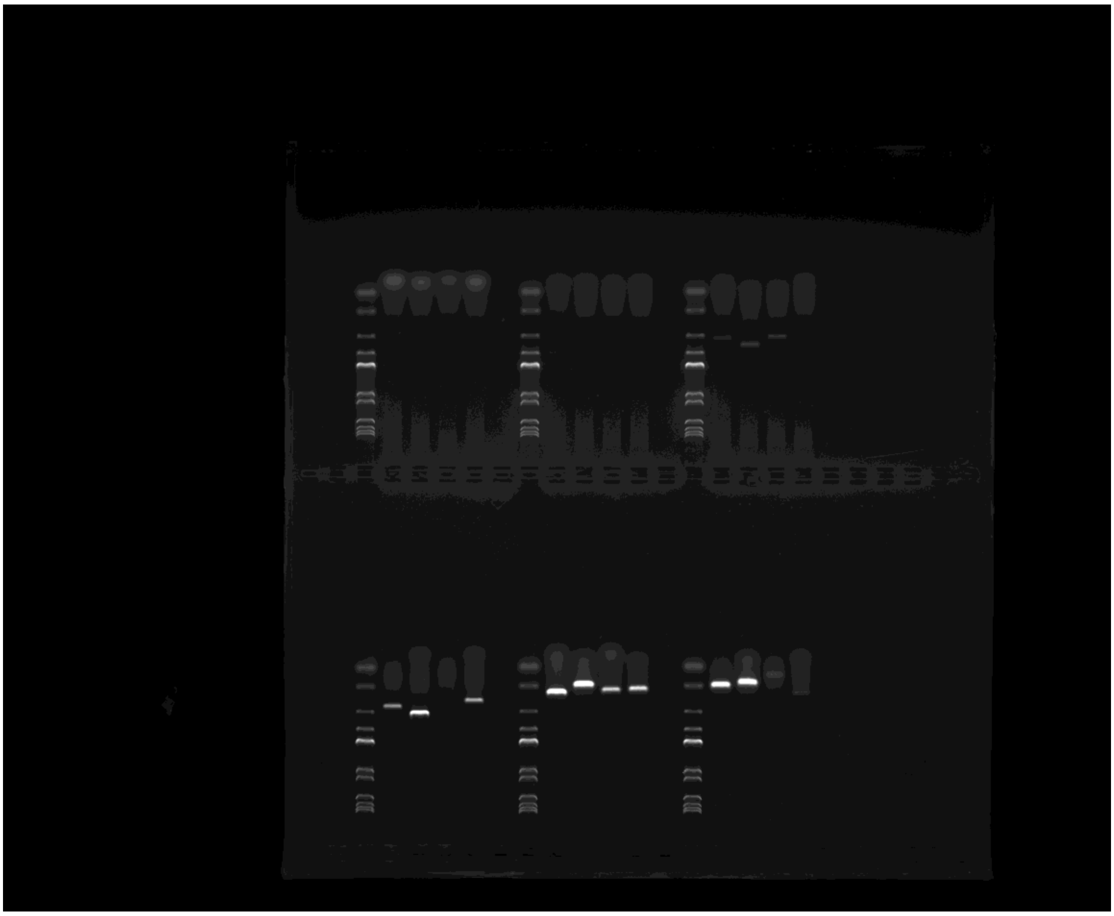

Figure S2. The raw Gel diagram of agarose gel electrophoresis. The bottom half of the image shows the results in Figure 2 of this study.
